# Phenotypic analysis method for 3D reconstruction of eggplant seedlings fused with background purification — based on the improved EggplantPointNet++ model and DBSCAN clustering

**DOI:** 10.3389/fpls.2026.1821217

**Published:** 2026-05-08

**Authors:** Jiamin Xu, Jinyao Dong, Ziyi Zhang, Yaben Zhang, Xiaofei Zhang, Chengxiao Deng, Junyi Li, Zhibo Zhong, Feng Pan, Hongbin Wu, Xiuqing Fu

**Affiliations:** 1 College of Engineering, Nanjing Agricultural University, Nanjing, China; 2 College of Smart Agriculture (College of Artificial Intelligence), Nanjing Agricultural University, Nanjing, China; 3 Institute of Farmland Water Conservancy and Soil-Fertilizer, Xinjiang Academy of Agricultural Reclamation Science, Shihezi, Xinjiang, China; 4 Institute of Mechanical Equipment, Xinjiang Academy of Agricultural Reclamation Science, Shihezi, Xinjiang, China

**Keywords:** 3D reconstruction, eggplant seedlings, EggplantPointNet++ model, phenotypic detection, point cloud segmentation

## Abstract

Eggplant (Solanum melongena L.) is a widely cultivated vegetable crop worldwide, occupying an important position in the agricultural industries of Asia, the Middle East, and Southern Europe. Its significance extends beyond agricultural economics to diverse dimensions such as dietary nutrition, rendering it of considerable research and application value. Traditional crop phenotyping methods suffer from low efficiency, substantial manual errors, and a tendency to damage tender seedlings, while existing three-dimensional phenotyping techniques face challenges including strong background interference and large data volumes. These dual constraints limit the accuracy and application feasibility of seedling phenotyping. To address these issues, this study proposes a non-destructive phenotyping method for eggplant seedlings, with the improvement of the PointNet++ architecture as its core and point cloud background purification as a key preprocessing step, aiming to enhance eggplant breeding efficiency and seedling screening accuracy. The raw point clouds first undergo background purification to actively remove seedling tray points, thereby improving point cloud purity and reducing data size. Concurrently, based on the PointNet++ model, we develop an improved point cloud segmentation model, EggplantPointNet++, by introducing multi-scale residual blocks, integrating channel attention mechanisms, incorporating a global context module, and refining the feature propagation layer. In conjunction with the DBSCAN clustering algorithm, this approach achieves semantic and instance segmentation of eggplant seedling point clouds, with certain improvements in segmentation accuracy and model efficiency under small-scale and occluded scenarios. To validate the technical effectiveness, multiple comparative experiments and ablation studies were conducted. The results demonstrate that EggplantPointNet++ outperforms the original model, background purification preprocessing provides positive gains, and each improved module contributes positively. The final model achieves improvements in core metrics including Recall and F1-score. Based on the segmented point cloud data, this study calculates core phenotypic parameters including plant height, stem diameter, cotyledon angle, and cotyledon area. Using the technical system established in this study, we completed the time-series measurement of three-dimensional morphological changes in eggplant seedlings during the cotyledon stage, providing quantitative references for seedling growth assessment and superior plant selection.

## 1 Introduction

### 1.1 Research background and significance of phenotypic detection in eggplant seedlings

Eggplant (Solanum melongena L.) is an annual herbaceous plant belonging to the genus Solanum in the family Solanaceae. It is also an important economic solanaceous crop widely cultivated worldwide, with rich nutritional value (Lyngdoh et al., 2025) and high economic benefits. The fruit growth period is approximately 6–8 months, and the fruit is rich in vitamins, anthocyanins, and unique phenolic antioxidant components (Lyu et al., 2024). As a major vegetable species in both protected agriculture and open-field cultivation, eggplant plays an important role in optimizing daily dietary structure and promoting the development of characteristic agricultural industries.

The growth vigor at the seedling stage is significantly positively correlated with the yield performance after transplanting. Therefore, seedling quality detection provides a fundamental guarantee for high yield in the later stage. Detection at the plant seedling stage can avoid transplanting inferior seedlings (such as weak-growing and morphologically abnormal seedlings) to the field, thus reducing later yield loss and ineffective management costs (Grossnickle and MacDonald, 2018).

However, current crop phenotyping studies mainly focus on staple food crops such as maize, wheat, and rice, while phenotyping research on solanaceous vegetables, especially eggplant seedlings, is relatively insufficient (Yang et al., 2020). Therefore, carrying out phenotyping detection of eggplant seedlings can provide technical support for high-quality seedling screening, intelligent seedling raising, and precise cultivation management of eggplant, which has important theoretical significance and application value for improving the modernization level of the eggplant industry.

### 1.2 Research status of crop phenotypic detection technologies

Crop phenotypic detection technologies are mainly divided into two-dimensional, three-dimensional, and multi-dimensional fusion technologies according to dimensionality. The use of two-dimensional phenotyping techniques for crop phenotyping detection is a common practice in agriculture. Local feature information of plants can be extracted from two-dimensional images (Yuan et al., 2024), such as the length, width, and area of leaves, as well as phenotypic information including plant height obtained from the front view (Zermas et al., 2020). Tang et al. proposed an accurate method for detecting tomato leaf diseases based on two-dimensional images. This method introduced an attention mechanism into the PLPNet network to eliminate the influence of soil background on disease detection, thereby improving the accuracy and specificity of the detection process (Tang et al., 2023). Jin et al. proposed a seedling selection and transplantation method for lettuce seedlings based on the ResNet18 network, which screened two-dimensional images of seedlings and achieved a screening accuracy of 97.44% in tests. After removing unhealthy seedlings, the survival rate of transplantation was effectively improved (Jin et al., 2022).

However, in practical scientific research and production, traditional phenotyping methods based on two-dimensional images are susceptible to shooting angles and lighting conditions, and have obvious problems such as incomplete feature extraction. It is difficult to extract information such as leaf curling degree from a single two-dimensional image (Fang et al., 2018). At the cotyledon stage of eggplant seedlings, the cotyledons are slender, thin, and tender. Moreover, seedlings are short and densely grown, and overlapping and occlusion easily occur between cotyledons, making it difficult to fully capture and accurately characterize the morphology of cotyledons through two-dimensional imaging techniques.

Compared with two-dimensional technology, three-dimensional technology can effectively solve the problem of occlusion, obtain complete spatial structure, and accurately quantify three-dimensional morphological parameters of crops (Omia et al., 2026). As eggplant germplasm research has entered the stage of precise and three-dimensional phenotypic analysis, three-dimensional point cloud-based reconstruction technology has become an effective way to obtain the real morphological structure of plants (Li et al., 2025). Research focus has gradually shifted from traditional two-dimensional phenotyping to fine quantification and dynamic monitoring of three-dimensional spatial features. In this context, it is urgent to promote the upgrading and transformation from two-dimensional to three-dimensional technology, and the exploration of three-dimensional phenotyping technology has become a key link to achieve further agricultural intelligence and high-throughput phenotyping analysis (Li et al., 2026). Reji et al. used 3D LiDAR point cloud technology to perform deep learning prediction of plant height and crown width for three vegetable crops: tomato, eggplant, and cabbage. Morphological parameters were calculated through digital plant reconstruction, realizing dynamic monitoring of the growth cycle (Reji and Nidamanuri, 2024). Zhang, W. et al. applied deep learning to achieve three-dimensional branch segmentation of maize tassels and extract phenotypic parameters. They also developed an automatic maize tassel phenotyping analysis system that achieved complete branch segmentation based on the shortest path algorithm. The intersection over union (IoU), precision, and recall of the segmentation results were 96.29%, 96.36%, and 93.01%, respectively (Zhang et al., 2023).

Although three-dimensional point cloud technology has demonstrated substantial potential in crop phenotyping, several challenges persist in current research, as outlined below.

1. Severe background interference and limited segmentation accuracy. In high-throughput plant phenotyping (HTPP) systems, seedlings are usually cultivated in standard containers (such as plug trays and seedling boxes). The collected three-dimensional point cloud data inevitably contain background information such as container walls, substrate, and supports (Han et al., 2022). Such background noise is spatially mixed with seedling organs, making it difficult for segmentation models to accurately distinguish the boundary between crops and background when dealing with small-scale and irregularly shaped seedling targets, resulting in reduced accuracy of stem and leaf segmentation (Chen et al., 2021).
2. Large point cloud data leads to high computational and storage costs. Raw point clouds acquired through three-dimensional scanning typically contain hundreds of thousands or even millions of points, resulting in data volumes far exceeding those of two-dimensional images (Omia et al., 2026). In high-throughput phenotyping (HTPP) applications, frequent observations of large-scale samples generate massive point cloud data, which limits model inference speed, consumes substantial storage resources, and prolongs analysis time (Poorter et al., 2023).
  In summary, developing a three-dimensional point cloud preprocessing method that effectively removes background interference, reduces data volume, and maintains segmentation accuracy is of great significance for overcoming the current technical bottlenecks in eggplant seedling phenotyping and promoting the practical application of three-dimensional phenotyping technologies in HTPP systems.

### 1.3 Research objectives and contents

To address the limitations existing in current three-dimensional crop phenotyping methods, such as severe background interference, redundant point cloud data, low computational efficiency, and insufficient boundary extraction accuracy, this study developed an improved point cloud model named EggplantPointNet++ for instance segmentation of eggplant seedlings based on the PointNet++ semantic segmentation model. The specific research objectives and contents are as follows:

1. Construct a three-dimensional point cloud dataset of eggplant seedlings at the cotyledon stage. Collect 360° omnidirectional images of eggplant from germination to the cotyledon stage in full time series, and establish a 3D phenotypic dataset covering the cotyledon stage, providing a data foundation for subsequent model training and phenotypic analysis.
2. Propose a background purification preprocessing strategy and improve the PointNet++ semantic segmentation model. Aiming at the core problems including background interference and redundant invalid data, an optimized framework of “Background Purification – Semantic Segmentation – Instance Segmentation” is proposed: First, background purification is used to eliminate interference from culture containers and other background components, achieving accurate separation between crops and background and reducing data volume. Second, semantic segmentation is applied to distinguish stem and leaf point clouds. Finally, the DBSCAN clustering algorithm is adopted to perform instance segmentation on the semantic segmentation results, realizing independent identification of individual seedlings.
3. Develop a method for analyzing phenotypic traits from three-dimensional point clouds of eggplant seedlings using EggplantPointNet++, and evaluate its performance on the dataset established in this study under the defined experimental conditions.

## 2 Materials and methods

### 2.1 Experimental equipment

#### 2.1.1 Seedling cultivation system

**Figure 1:**
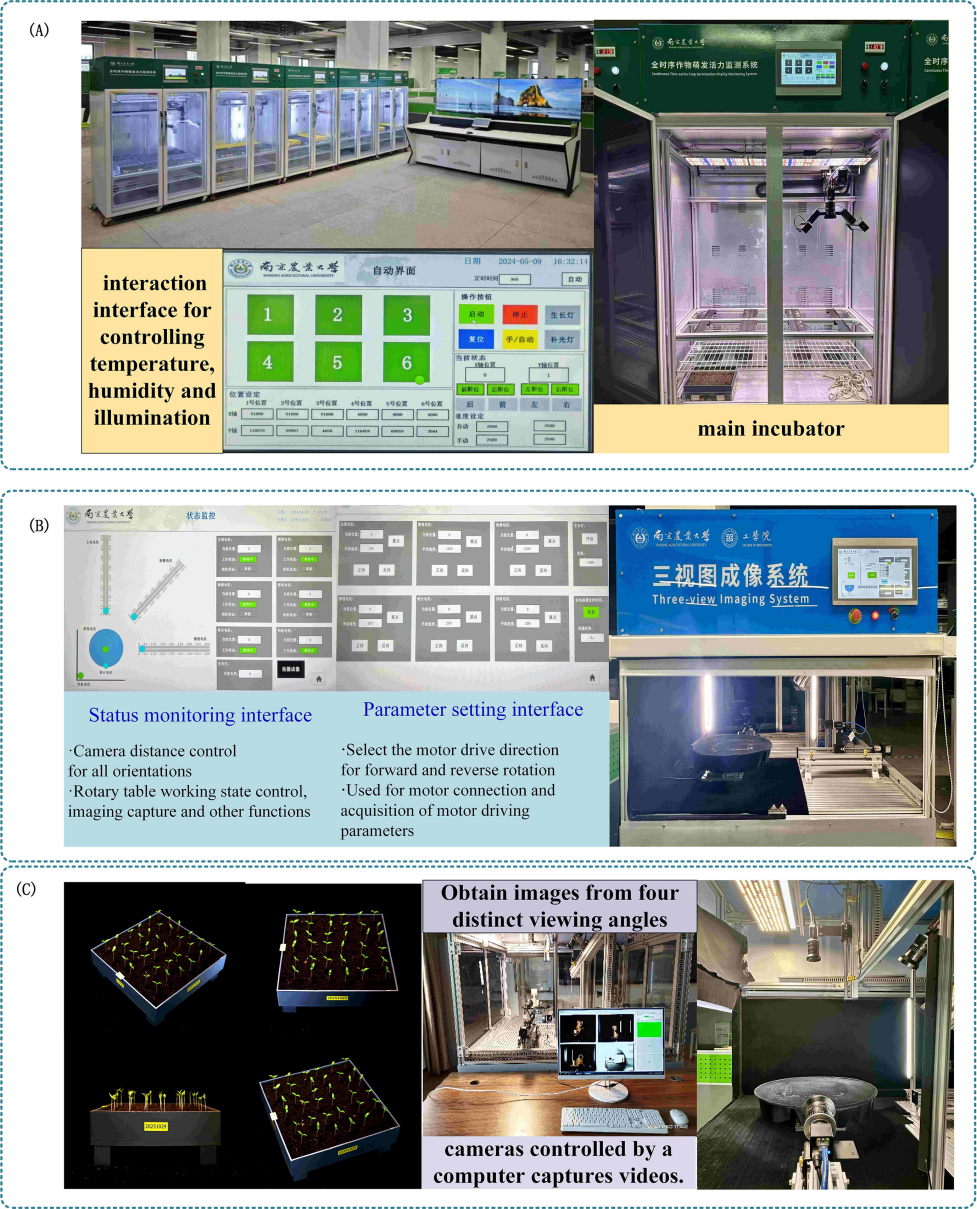
**(A)** Seedling incubator **(B)** Three-view imaging system **(C)** 3D image extraction process.

The seedling cultivation system adopted in this study integrates a seed cultivation module, an environmental control module, and a human–computer interaction module, as shown in **Figure 1A**. This system is equipped with an independent and adjustable temperature-light coupling control system, with a temperature control range of 10–50 °C. A dedicated full-spectrum LED array provides uniform illumination to ensure stability and consistency of the seedling growth environment. Six 3D-printed culture trays with a size of 25 cm × 25 cm can be placed inside the chamber simultaneously, enabling parallel cultivation of different experimental groups. The human–computer interaction function is realized through a touch screen installed on the chamber, which allows intuitive setting and regulation of key environmental parameters such as temperature, humidity, and light intensity.

#### 2.1.2 Three-view imaging system

To accurately obtain the three-dimensional spatial information of eggplant seedlings, this study adopted a three-view imaging system based on 3D data reconstruction for crop phenotyping. Its structural design is shown in **Figure 1B** (Han et al., 2025). The system is mainly composed of a frame structure, imaging sensing device, motion module, acquisition platform, and control panel. It enables multi-angle and omnidirectional image capture of plants from four views: front, side, top, and axonometric, as illustrated in **Figure 1C**.

### 2.2 Seedling growth and data acquisition

To establish a 3D point cloud model of eggplant seedlings, 300 seeds of Jingnongyan Zihong Changqie (purchased from Beijing Academy of Agriculture and Forestry Sciences Seed Industry) with uniform size and intact morphology were selected in this study. Appearance screening and size calibration were performed to ensure the reliability and repeatability of experimental data.

The seeds were soaked in deionized water at 30 °C for 8 hours to activate cellular metabolism and ensure optimal water content. After soaking, full seeds sinking to the bottom of the container were collected and evenly spread on a sterilized wet towel. They were then placed in the aforementioned germination chamber and primed for 24 hours at a constant temperature of 28 °C under continuous light to regulate the physiological state of the seeds and guarantee maximum germination vigor.

**Figure 2:**
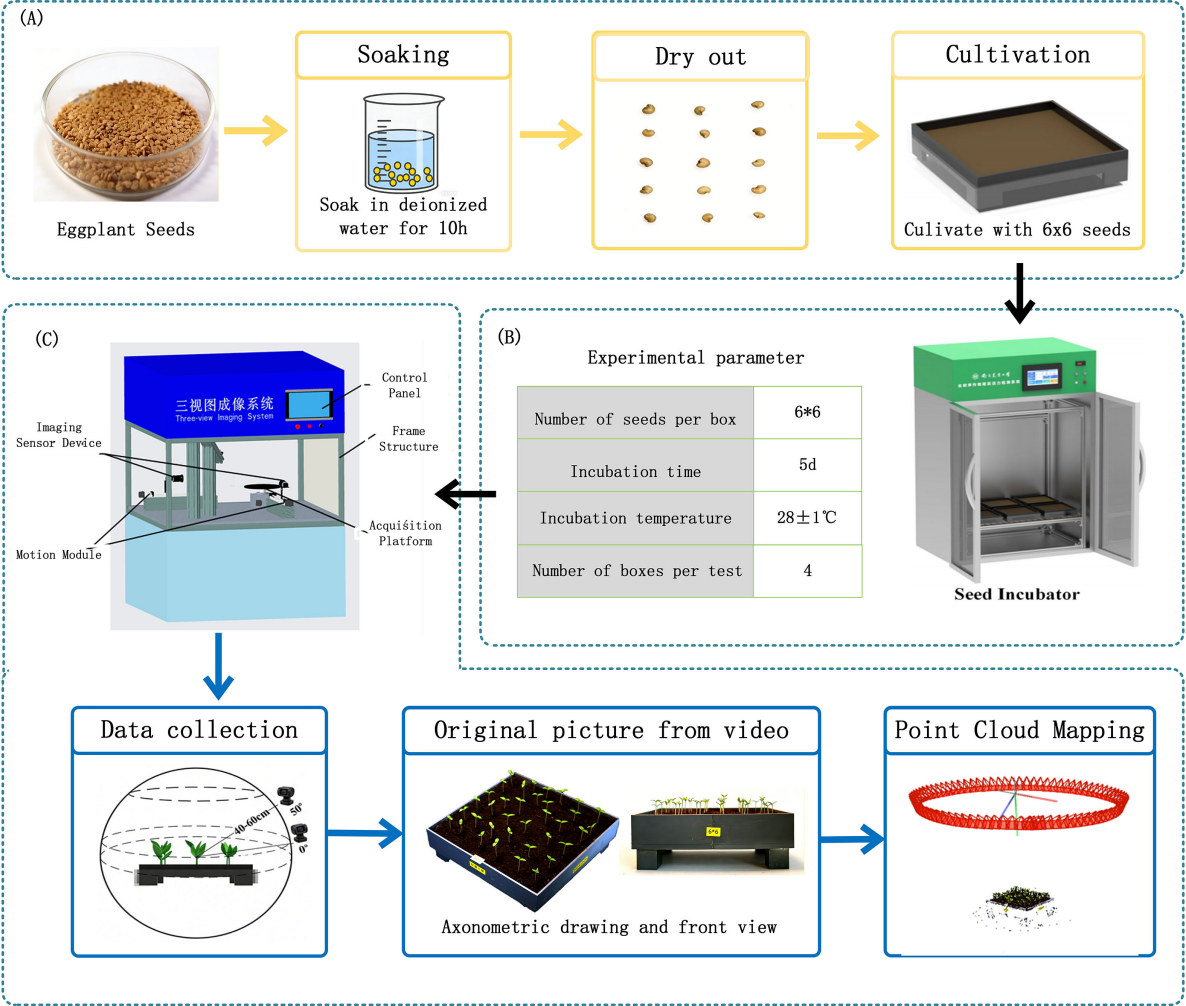
**(A)** Seed priming **(B)** Constant temperature and humidity incubator **(C)** 3D imaging and reconstruction.

After priming, 144 healthy and full eggplant seeds with similar germination status were selected and arranged in a 6×6 grid evenly in 4 soil cultivation trays. The trays were placed at 4 fixed positions in the seed growth and cultivation module, with a spacing of 41 mm between each seed, as shown in **Figure 2A**. Continuous cultivation was conducted for 5 days.

During the experiment, environmental parameters including temperature, humidity, and light in the germination chamber were strictly controlled according to preset standards to ensure the stability and consistency of the growth environment for eggplant seedlings. The specific experimental parameters are presented in **Figure 2B**.

After seedling emergence, the multi-angle imaging system for crop phenotypic 3D reconstruction was employed to collect video data of seedlings in individual trays at 24-hour intervals. To mitigate the interference of leaf occlusion on 3D reconstruction, the midpoint of the height of the central seedling in the tray was set as the focal point, with the shooting distance maintained at 40–60 cm. A 360° rotational shoot was performed from two specific angles relative to the ground: 0° (front view) and 50° (axonometric view), ensuring full coverage of the seedlings, as illustrated in **Figure 2C**.

**Figure 3:**
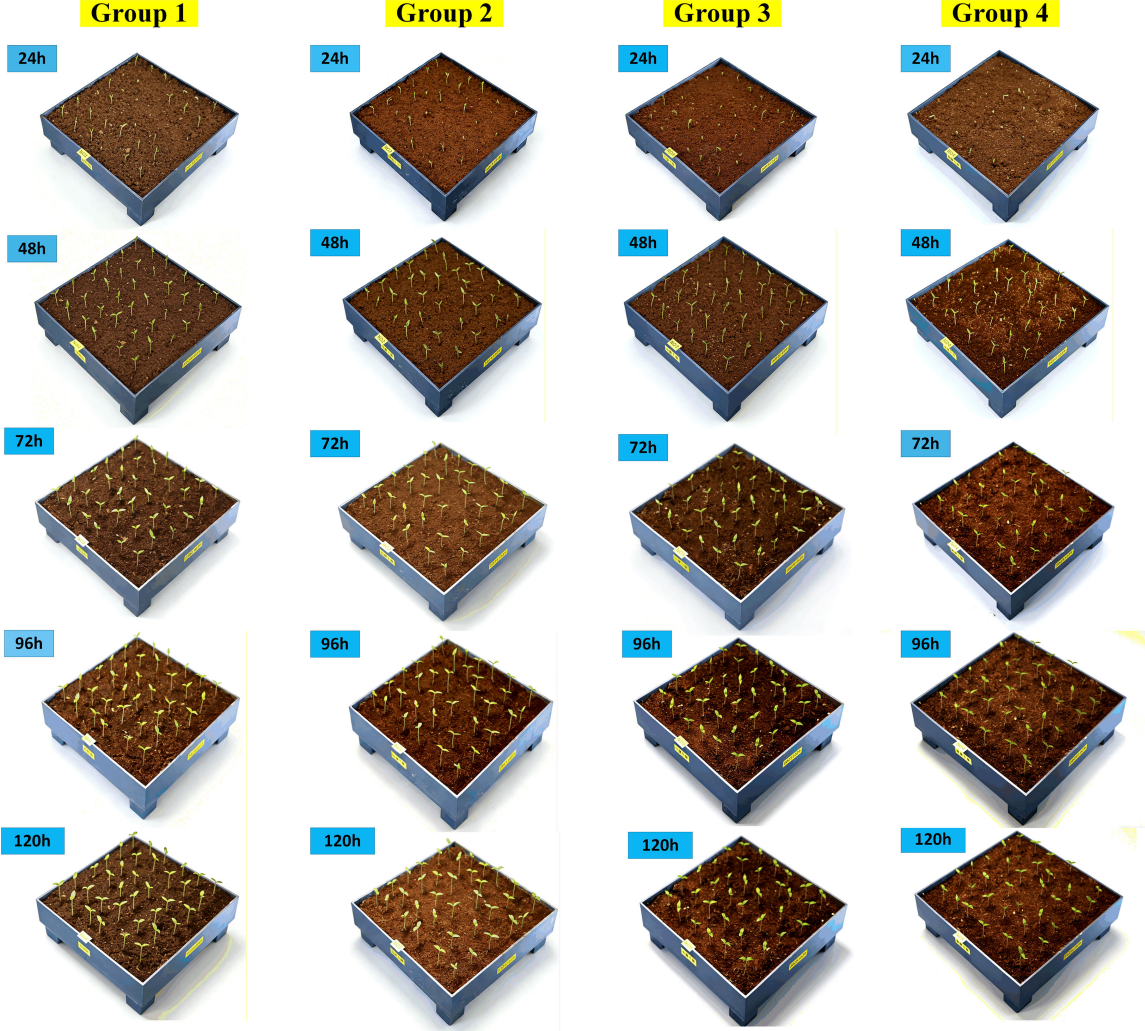
Full-temporal eggplant data images.

Each video lasted approximately 2 minutes. Subsequently, frame extraction technology was applied to capture one static image every 4°, yielding approximately 90 images per video. Ultimately, a total of 1,800 high-quality static images were retained, serving as the foundational dataset for the 3D point cloud modeling of eggplant seedlings as shown in **Figure 3**.

### 2.3 Dataset construction

#### 2.3.1 3D reconstruction

In this study, the COLMAP software was used to perform 3D reconstruction from the collected static images of eggplant seedlings. Based on the core algorithms of Structure from Motion (SfM) and Multi-View Stereo (MVS), this software realizes the construction of a 3D point cloud model of seedlings through a three-step pipeline: feature extraction, pose estimation, and dense reconstruction.

Based on the 1,800 collected static images of eggplant seedlings, the SIFT algorithm built into COLMAP was used to extract image feature points. This algorithm can effectively capture key features such as cotyledon edges and textures in the images, generating feature descriptors with scale invariance and rotation invariance.

Based on the optimized feature matching results, COLMAP uses Structure from Motion (SfM) to solve the intrinsic and extrinsic parameters of the camera and complete camera pose estimation, accurately restoring the spatial position and shooting angle of the camera during image acquisition. The 3D spatial point coordinates are calculated via the triangulation algorithm to generate an initial sparse point cloud model, which roughly reflects the spatial structure outline of eggplant seedlings and provides initial geometric constraints for subsequent dense reconstruction (Schönberger and Frahm, 2016).

Subsequently, to address the lack of detailed information in the sparse point cloud model, the Multi-View Stereo (MVS) algorithm in COLMAP was adopted for dense reconstruction. Through depth estimation and fusion of pixel-level information for each image, the missing spatial details in the sparse point cloud are supplemented, generating a dense point cloud model with higher density and richer details (Yao et al., 2018).

After reconstruction, post-processing operations such as denoising were performed on the dense point cloud to further improve the quality of the point cloud model. Finally, a 3D point cloud model of eggplant seedlings that can be used for subsequent phenotypic parameter extraction was obtained.

#### 2.3.2 Coordinate alignment and scale calibration

To ensure the accuracy of phenotypic parameter calculation, it is necessary to first perform coordinate alignment and scale calibration on the segmented eggplant seedling point cloud to eliminate deviations introduced during 3D reconstruction. The RANSAC algorithm (Fischler and Bolles, 1981) was used to detect the ground plane and obtain its normal vector. The angle between this normal vector and the target Z-axis (perpendicular to the ground) was calculated, and the rotation matrix was solved via Rodrigues’ rotation formula to align the point cloud Z-axis perpendicular to the ground, thus completing coordinate alignment (Yang et al., 2024).

Using the eggplant seedling cultivation tray as the reference object, the scale factor was computed based on the ratio between the actual side length of the tray and the corresponding length in the 3D reconstruction model, achieving accurate correspondence between the point cloud data and real physical dimensions. The formula is as follows:


$$\text{k}=\frac{{\text{L}}_{\text{real}}}{{\text{L}}_{\text{virtual}}}$$

Where, k is the scale factor, ${\text{L}}_{\text{real}}$ is the actual side length of the culture tray, and ${\text{L}}_{\text{virtual}}$ is the corresponding side length of the tray in the 3D reconstruction model.

#### 2.3.3 Data annotation

After acquiring the 3D point cloud data of eggplant seedlings, each eggplant seedling data file was finely annotated for model training. In this study, CloudCompare software (Martinez-Guanter et al., 2019) was used to annotate the point clouds of eggplant seedlings at different growth stages. The annotated data were saved in.txt format, with each line corresponding to one point, including x, y, z 3D coordinates, r, g, b color information, Nx, Ny, Nz normal vectors, and two labels (label1 and label2). Among them, label1 was used for semantic segmentation: 0 represented pot point cloud, 1 represented stem point cloud, and 2 represented leaf point cloud; label2 was used for instance segmentation verification: 0 represented pot point cloud, and positive integers such as 1, 2, and 3 corresponded to the stems and leaves of individual eggplant seedling instances respectively, realizing accurate distinction between stems and leaves of different seedling individuals.

#### 2.3.4 Background purification

After completing the annotation of eggplant seedling point clouds, this study performed targeted background purification as a core step in point cloud data preprocessing. A semi-automated workflow based on the annotation results described in Section 2.3.3 was adopted, utilizing custom Python scripts to achieve efficient background removal. This approach balances automation efficiency with manual verification accuracy, reducing the errors and inefficiencies associated with fully manual operations.

The primary targets of background purification were the seedling tray point clouds with a label value of 0. Such background points account for a very high proportion of the entire dataset, with a single sample containing up to over one hundred thousand points, representing redundant data that interferes with model feature learning. The semi-automated purification workflow operated on the.txt format files generated from previous annotations. The custom-developed Python script directly read the annotated point cloud data and automatically filtered out and removed all seedling tray background points with a label value of 0.

**Figure 4:**
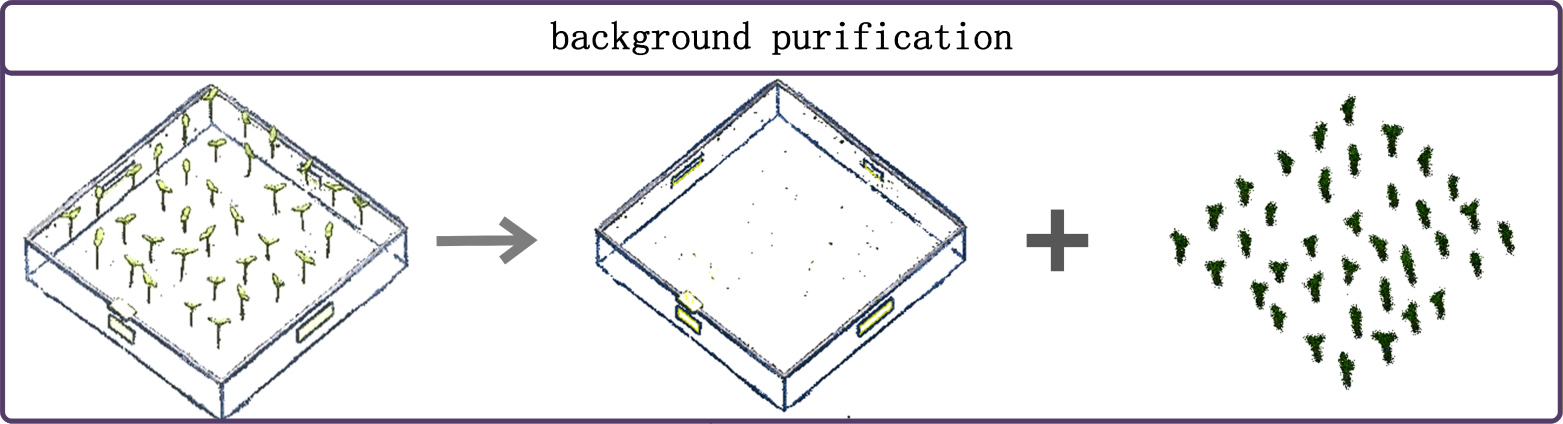
Example of background purification.

After script execution, new purified point cloud files were automatically generated, retaining only the valid plant data with label values of 1 (stem points) and 2 (leaf points), thereby effectively reducing interference from redundant background (as shown in **Figure 4**). In accordance with standard academic data retention practices, the original annotated source point cloud data were fully preserved to facilitate subsequent data tracing, replication, and error analysis.

Through the semi-automated background purification process described above, over one hundred thousand invalid background points were removed per sample, significantly reducing the size of the point cloud dataset and the computational burden of model training and inference. This effectively improved data processing efficiency, model training speed, and inference performance, providing high-quality data support for the subsequent lightweight deployment of the model on agricultural inspection terminal devices. The resulting high-purity point cloud dataset, with its data dimensions and feature distribution, is better suited to the training requirements of point cloud segmentation models and also provides a high-quality data foundation for subsequent preprocessing steps such as data augmentation and format conversion (Li et al., 2024). 

As shown in **Table 1**, the number of box point clouds in each region ranged from 130,000 to 190,000, whereas the numbers of leaf and stem point clouds were only several thousand to tens of thousands. The box point clouds generally accounted for more than 75% of the total point clouds in each region, which directly verified the massive volume of background box points and the extreme imbalance between foreground and background data.

**Table 1:** Statistics of point cloud quantity in different regions (Unit: point).

Area	Box	Leaf	Stem
Area1	134428	6580	1431
Area2	186279	13303	6347
Area3	184979	14014	9796
Area4	135755	37838	26300
Area5	184056	37024	25337
Area6	158412	28587	18446
Area7	152062	30133	21941
Area8	137629	48736	37287

This data characteristic further highlights the technical necessity of background purification. Removing the highly redundant background point clouds can effectively improve data quality and computational efficiency, clearing key obstacles for subsequent model training and segmentation tasks.

#### 2.3.5 Dataset partitioning

To ensure the objectivity and reproducibility of model evaluation and to strictly avoid data leakage, the complete dataset was partitioned into training, validation, and test sets at a ratio of 7:1:2, with individual pots serving as the basic partitioning unit. All point cloud samples corresponding to each pot, including those acquired at different time points, were assigned entirely to the same subset, ensuring that data from the same pot did not appear across multiple subsets. A random partitioning strategy was adopted to mitigate overly optimistic estimates of generalization performance that may arise from specific data distribution biases, thereby providing a more realistic assessment of model generalizability on unseen data. The training set was used for model parameter learning, the validation set for hyperparameter tuning, and the test set for final performance evaluation. To enhance the statistical reliability of the evaluation results, all experiments were conducted with three independent runs. For each run, the random partitioning and model training were repeated, and all evaluation metrics are reported as the mean and standard deviation across the three runs.

#### 2.3.6 Data augmentation

To enhance the robustness and generalization capability of the network model, data augmentation was applied exclusively to the original point clouds in the training set to increase the number and diversity of training samples. The validation set and test set were kept in their original state without any augmentation operations to ensure the authenticity and reliability of the evaluation results. The augmentation techniques employed are shown in **Table 2**: 

**Table 2:** Details of data augmentation methods.

Augmentation method	Description	Parameter range
Fake Dropout	Randomly remove partial points	10%–20%
Jittering	Add Gaussian noise	σ = 0.01, range ±0.05
Rotation	Rotate independently around X/Y/Z axes	± 10°
Scaling	Randomly scale the point cloud	0.7–1.3
Shuffling	Randomly reorder point indices	Random permutation
Translation	Random shift along X/Y/Z axes	± 0.1

A total of 90 point cloud files (including the original data) were generated after augmentation.

In this study, the parameters of each data augmentation operation were meticulously recorded. In particular, for the scaling factors used in the scaling operation, an association of “sample ID – augmentation type – parameter value” was established. This recording mechanism ensures that when extracting phenotypic parameters (e.g., stem diameter, plant height, and leaf area) of eggplant seedlings in subsequent steps, the corresponding scaling factor of each sample can be accurately retrieved to restore the scaled point cloud data to their original physical dimensions.

Furthermore, recording the parameters of other augmentation operations (e.g., rotation and translation) can assist in correcting spatial position deviations during feature extraction. This prevents phenotypic parameter calculation errors caused by data augmentation from the perspective of data traceability, thereby ensuring the accuracy and repeatability of phenotypic feature quantification results.

#### 2.3.7 Dataset format conversion

After data augmentation was completed, all point cloud data were uniformly converted into the 3SDIS standard format to meet the input requirements of the PointNet++ model. During the conversion process, the file organization specifications of the 3SDIS dataset were strictly followed. Primary subdirectories were created according to Area, and secondary subdirectories were established under each Area based on different growth scenarios. For each scenario, the point cloud files and label files were merged, integrating complete information including x, y, z coordinates, Nx, Ny, Nz normal vectors, label1 (semantic segmentation labels), and label2 (instance segmentation labels), thereby eliminating data redundancy. To accelerate data reading speed during model training, the merged data for each scenario were uniformly saved in.npy format, leveraging its binary storage characteristics to improve data loading efficiency.

## 3 Optimization of PointNet++ point cloud segmentation model

As a representative network in 3D point cloud processing, PointNet++ enables effective processing of unordered point cloud data through hierarchical feature learning and local geometric structure encoding, and exhibits favorable performance in point cloud semantic segmentation tasks (Qi et al., 2017). The architectural characteristic of this network that directly processes raw point clouds allows it to maintain stable feature extraction capabilities across various scenarios. However, when dealing with special point clouds of eggplant seedlings characterized by tiny organs, dense structures, and severe occlusion, the original PointNet++ network still faces several challenges: limited perception capability for fine local geometric features such as leaves and stems, insufficient modeling of long-range dependencies within the overall plant structure, and high sensitivity to the extreme class imbalance between foreground and background point clouds. These issues compromise the accuracy and robustness of the network in fine-grained structural segmentation to a certain extent.

Among existing peer point cloud segmentation models, DGCNN (Wang et al., 2019) captures local geometric correlations of point clouds based on graph convolution and delivers excellent performance in general-scenario segmentation, yet it exhibits poor adaptability to the sparsity and small-target features of agricultural point clouds. It is prone to gradient vanishing when extracting features of slender stems and thin cotyledons of eggplant seedlings, and its high computational complexity makes it incompatible with the efficiency requirements of high-throughput agricultural detection. PointCNN (Li et al., 2018) addresses the disorder of point clouds via X-transformation and possesses strong fine-grained segmentation ability, but its feature modeling capability for occluded scenarios is weak, failing to effectively distinguish the boundaries of overlapping cotyledons in eggplant seedlings; additionally, it lacks targeted optimization for the foreground-background class imbalance problem in agricultural scenarios (Fareed et al., 2023). As a dedicated improved model for agricultural phenotyping, PlantPointNet (Li et al., 2022) is adaptable to crop seedling segmentation scenarios, but it only introduces a single spatial attention mechanism, lacks modeling of long-range dependencies for the overall plant structure, and cannot fully capture the stem-leaf topological correlations of eggplant seedlings.

DRP-Net (Liu et al., 2024), a specialized model for solanaceous seedling segmentation, achieves accurate stem-leaf segmentation of tomatoes but focuses on dynamic feature transfer across growth stages, resulting in insufficient capture of local features for extremely tiny organs at the cotyledon stage. Moreover, it does not integrate background purification preprocessing, leading to a significant drop in segmentation accuracy under the interference of nursery box background. MS-PCNet (Zhang et al., 2023) optimizes agricultural small-target segmentation based on multi-scale convolution but does not incorporate global context modeling, limiting its ability to distinguish organ boundaries in densely occluded regions of eggplant seedlings. FPN-Point (Sun and Li, 2024) enhances overall plant segmentation performance by integrating a feature pyramid network, but it suffers from a large number of parameters (approximately 8.2M) and slow inference speed, failing to meet the real-time demands of high-throughput detection.

**Figure 5:**
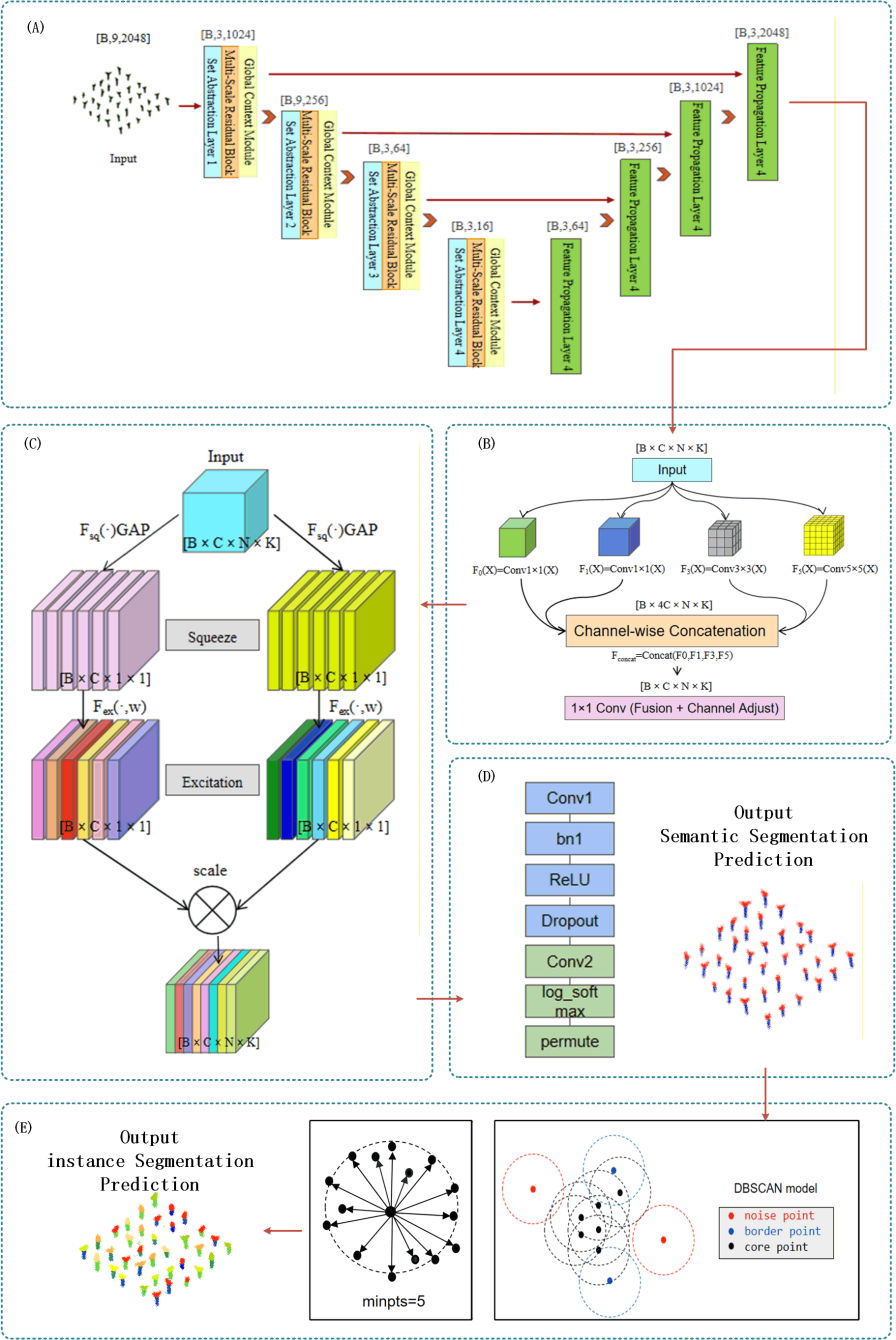
**(A)** Feature extraction and feature fusion module **(B)** Multi-scale convolution module **(C)** Channel attention mechanism module **(D)** Improved module integration and output **(E)** DBSCAN clustering algorithm and instance segmentation module.


To address the above challenges, this study made targeted improvements based on the PointNet++ framework and proposed the EggplantPointNet++ model, which effectively improved the semantic segmentation performance on complex seedling point clouds (as shown in **Figure 5A**). To make up for the deficiency in local feature perception, we introduced a multi-scale convolution module to enhance the ability to capture small geometric structures such as leaf edges and stem details (Wang et al., 2019); to improve long-range context modeling, the model added a global context module to strengthen the understanding of the overall plant morphology and the dependency relationships between organs; at the same time, the channel attention mechanism was used to optimize feature selection, suppress background noise, and alleviate the impact caused by class imbalance (as shown in **Figure 5D**). In addition, residual connections were embedded in key layers of the model to further stabilize the training process and promote gradient flow.

### 3.1 Core improved modules

#### 3.1.1 Multi-Scale Residual Block

##### 3.1.1.1 Multi-scale convolution module design

The original PointNet++ mainly relies on Multi-Layer Perceptron (MLP) and non-linear transformation to extract point features, with a relatively single perception scale for local geometric structures. The stems (thin cylinders) and leaves (thin curved surfaces) of seedlings exhibit distinct local patterns in point clouds. Therefore, to extract richer geometric information of local neighborhoods, we designed a multi-scale convolution module. Given the input point cloud feature ${\text{F}}_{\text{in}}\in {\mathbb{R}}^{\text{N}\times {\text{C}}_{\text{in}}}$, where N is the number of points and ${\text{C}}_{\text{in}}$ is the number of input channels, the module applies convolution operations with kernel sizes K = 1, 3, 5 in parallel, as shown in **Figure 5B**:

$${\text{F}}_{\text{k}}={Conv1D}_{\text{K}=\text{k}}({\text{F}}_{\text{in}}),\text{k}\in \{1,3,5\}$$

Among them, convolution with k=1 extracts point-level features, convolution with k=3 captures local neighborhood patterns, and convolution with k=5 obtains contextual information in a larger range. The multi-scale features are concatenated and then fused through 1×1 convolution:

${\text{F}}_{concat}=\text{Concat}({\text{F}}_{1},{\text{F}}_{3},{\text{F}}_{5})$


$${\text{F}}_{ms}={Conv1D}_{1\times 1}({\text{F}}_{concat})$$

##### 3.1.1.2 Channel attention mechanism (squeeze-and-excitation block)

Different channels of point cloud features may correspond to different importance of geometric or color information. To further improve the representation efficiency of features, we embedded a channel attention module after multi-scale convolution. In seedling scenarios, feature channels related to stem direction or leaf surface normal may be more discriminative than certain color channels. We introduced a lightweight channel attention module (SE Block), whose workflow is shown in **Figure 5C** (Hu et al., 2018).

Channel Weight Calculation Formula:

$\text{s}=\sigma ({\text{W}}_{2}\delta ({\text{W}}_{1}\text{z}))$


$${\text{F}}_{out}=\text{s}\cdot {\text{F}}_{in}$$

Among them, z is the feature vector after global average pooling, ${\text{W}}_{1}$ and ${\text{W}}_{2}$ are the weights of two fully connected layers, δ is the ReLU activation function, σ is the Sigmoid activation function, and · denotes channel-wise multiplication. This mechanism enables the network to adaptively calibrate channel feature responses, enhance the focus on key channels for segmentation tasks, and suppress redundant or noisy channels.

##### 3.1.1.3 Residual connections

To avoid gradient vanishing or gradient degradation that may occur during the training of deep networks, and to promote the effective reuse of multi-level features, this study introduced a Residual Connection mechanism in each enhanced feature extraction block. The basic form of the residual connection is shown in the formula:


$${\text{F}}_{output}=H({\text{F}}_{input})+F({\text{F}}_{input})$$

Among them, H(·) represents the main branch (including multi-scale convolution and attention), and F(·)represents the residual mapping. When the input and output dimensions are consistent, an identity mapping is adopted. This ensures that even if the network is deepened, the important geometric information at the bottom layer can be effectively propagated to the high layer.

#### 3.1.2 Global context module

To make up for the limitations of local convolution and attention operations in modeling long-range dependency relationships, this study introduced a lightweight Global Context Module at the end of the (encoder/decoder) (Vaswani et al., 2017). This module aims to inject the structural information of the entire point cloud scene as contextual clues into the feature representation of each point, thereby enhancing the model’s inference ability in complex situations such as occlusion and sparsity (Wang et al., 2023).

Specifically, the module first extracts scene-level feature descriptors through Global Average Pooling (GAP):


$$\text{g}=\frac{1}{N}\sum_{i=1}^{N}{\text{F}}_{\text{in}}(i,:)$$

Among them, $\text{g}\in {\mathbb{R}}^{1\times C}$ aggregates the channel statistical information of all points. Subsequently, the global information is compressed, refined and restored through a Bottleneck structure—i.e., two fully connected layers (or 1×1 convolutions) and non-linear activation functions:


$${\text{g}}^{\prime }={\text{W}}_{u}\cdot \delta ({\text{W}}_{d}\cdot \text{g})$$

Here, ${\text{W}}_{d}\in {\mathbb{R}}^{\frac{C}{r}\times C}$is the dimensionality reduction layer, ${\text{W}}_{u}\in {\mathbb{R}}^{C\times \frac{C}{r}}$ is the recovery layer, r>1 is the compression ratio, and δ is the ReLU activation function. This design can effectively model the non-linear dependencies between channels and suppress noise.

Finally, the refined global context vector $\;{\text{g}}^{\prime }$ is added point-wise to the original input feature ${\text{F}}_{\text{in}}$ through the Broadcast mechanism to form the final enhanced feature:


$${\text{F}}_{\text{out}}={\text{F}}_{\text{in}}+\text{Broadcast}({\text{g}}^{\prime })$$

With minimal computational overhead, this operation enables the local features of each point to “perceive” the global morphological structure of the entire seedling. This is particularly important when dealing with severely occluded areas. Based on the understanding of the overall structure (such as the growth direction of stems and the distribution law of leaves), the model can make more reasonable semantic inferences for locally missing points.

#### 3.1.3 Improved feature propagation layer

In the original PointNet++ model, the feature propagation layer realizes feature upsampling through nearest neighbor interpolation and feature concatenation. To enhance the expression and refinement ability of this layer in the feature recovery process, this study improved the feature propagation layer: after completing feature interpolation and concatenation, a multi-scale residual block is introduced to deeply process the fused features, followed by the final feature mapping. The operation process of the improved feature propagation layer is as follows:

1. Feature Interpolation: From the low-level feature map ${\text{F}}_{l}\in {\mathbb{R}}^{N_{l}\times C_{l}}$,the features at the high-level point positions are restored through the k-nearest neighbor inverse distance weighted interpolation algorithm to obtain the initial upsampled feature ${\hat{\text{F}}}_{h}\in {\mathbb{R}}^{N_{h}\times C_{l}}$.
2. Feature Concatenation: The interpolated feature ${\hat{\text{F}}}_{h}$ is concatenated with the skip connection feature ${\text{F}}_{h}^{\text{skip}}\in {\mathbb{R}}^{N_{h}\times C_{h}}$ from the corresponding layer of the encoder along the channel dimension to obtain the preliminary fused feature ${\text{F}}_{\text{fuse}}\in {\mathbb{R}}^{N_{h}\times (C_{l}+C_{h})}$.
3. Feature Enhancement: ${\text{F}}_{\text{fuse}}$ is fed into the multi-scale residual block for deep feature refinement and multi-scale information fusion to obtain the enhanced feature ${\text{F}}_{\text{enhanced}}$.
4. Feature Mapping: Finally, a standard 1×1 convolution layer is used to map the number of channels to the target dimension, and the final upsampled feature ${\text{F}}_{\text{out}}^{\text{FP}}\in {\mathbb{R}}^{N_{h}\times C_{\text{out}}}$ is output.
  This process can be formally expressed as:

$${\text{F}}_{\text{out}}^{\text{FP}}={Conv1D}_{1\times 1}(\text{MultiScaleResBlock}(\text{Concat}(\text{Interp}({\text{F}}_{l}),\;{\text{F}}_{h}^{\text{skip}})))$$

Through the introduction of a feature processing unit with multi-scale perception and residual learning capabilities, this improved mechanism significantly enhances the ability to recover detailed structures and local geometric patterns during upsampling, thereby improving the model’s discriminative performance on boundaries and small-scale structures in segmentation tasks.

#### 3.1.4 Loss function and optimization strategy

To maintain fairness in comparison with the baseline method, this study adopted the cross-entropy loss function used by PointNet++ (Goodfellow et al., 2016). Let the predicted category score of the network for the point cloud be $\text{P}\in {\mathbb{R}}^{N\times K}$, where N is the number of points and K is the number of categories; the true label is Y, then the loss function is defined as:


$$L=-\frac{1}{N}\sum_{i=1}^{N}\sum_{k=1}^{K}1\{y_{i}=k\}\text{log}\frac{\exp(P_{ik})}{\sum_{j=1}^{K}\text{exp}(P_{ij})}$$

In the formula, 1{·} is the indicator function, which takes a value of 1 when the condition in the parentheses is satisfied, otherwise 0.

In addition, the training strategies of the improved model (including hyperparameters such as optimizer type, learning rate scheduling strategy, batch size and number of iterations) are consistent with the baseline model, ensuring that the performance improvement comes only from the architectural improvements proposed in this study, not from differences in training configurations. This study adopted the Adam optimizer (Kingma and Ba, 2015) for model training, which has shown good convergence and stability in deep learning tasks.

In summary, by systematically integrating advanced mechanisms including multi-scale perception, channel attention, residual connections and global context modeling, the EggplantPointNet++ model constructs an efficient and robust deep learning framework specifically tailored for the 3D point cloud segmentation task of eggplant seedlings, laying a solid technical foundation for subsequent automated and precise extraction of plant phenotypic parameters (e.g., stem length, leaf area, and spatial distribution of organs).

### 3.2 Model training and evaluation metrics

#### 3.2.1 Model training configuration

The training and testing of the model were conducted in a standard computing environment, The specific configurations are shown in **Table 3**. 

**Table 3:** Configuration of the experimental simulation system.

Computing environment	Operating system	Ubuntu22.04
	Processor	Intel Xeon Gold 6248R @ 3.00GHz
	Memory	80 GB
	GPU Acceleration	4090
Framework & Language	Deep Learning Framework	PyTorch 2.4
	Programming Language	Python 3.10
Training Hyperparameters	Total Epochs	150
	Batch Size	16
	Optimizer	Adam
	Initial Learning Rate	0.001
	Weight Decay	1e-4
	Learning Rate Scheduling	Step decay; decayed to 0.5× the original value every 10 epochs
Data Preprocessing	Point Cloud Sampling	Input point clouds uniformly resampled to 2049 points

#### 3.2.2 Model evaluation metrics

To comprehensively and accurately evaluate the semantic segmentation performance of the EggplantPointNet++ model, four core evaluation metrics widely recognized in the field of 3D semantic segmentation were adopted in this study. These metrics quantify model performance from different dimensions and can fully reflect the model’s performance in the eggplant seedling point cloud segmentation task. The definitions and calculation formulas of each metric are as follows:

##### 3.2.2.1 Precision

Precision measures the proportion of points predicted as positive classes (e.g., leaves, stems) by the model that are actually positive classes, reflecting the accuracy of the prediction results. This metric focuses on the reliability of the model’s prediction outcomes (Everingham et al., 2010).


$$\text{Precision}=\frac{TP}{TP+FP}$$

Where:

TP (True Positive): The number of points correctly predicted as positive classes by the model.

FP (False Positive): The number of points incorrectly predicted as positive classes by the model (negative class points misclassified as positive classes).

##### 3.2.2.2 Recall

Recall measures the proportion of actual positive class points that are correctly predicted by the model, reflecting the model’s ability to detect positive class targets. This metric focuses on the coverage of real targets by the model (Long et al., 2014).


$$\text{Recall}=\frac{TP}{TP+FN}$$

Where:

FN (False Negative): The number of points incorrectly predicted as negative classes by the model (positive class points missed in prediction).

##### 3.2.2.3 F1 score

The F1 Score is the harmonic mean of precision and recall, which can comprehensively measure the model’s balanced performance between accuracy and completeness. This metric is particularly suitable for scenarios where there is a significant imbalance in the number of foreground and background points (Lipton et al., 2014).


$$\text{F}1=2\times \frac{\text{Precision}\times \text{Recall}}{\text{Precision}+\text{Recall}}$$

##### 3.2.2.4 Intersection over Union and mean Intersection over Union

Intersection over Union, also known as the Jaccard Index, is one of the most core metrics in semantic segmentation tasks. It calculates the ratio of the intersection to the union of the model’s predicted region and the ground-truth annotated region, directly reflecting the degree of overlap between the segmentation boundary and the real region (Eelbode et al., 2020).


$$\text{IoU}=\frac{\text{T}P}{\text{TP}+\text{FP}+\text{FN}}$$

To provide a comprehensive assessment of model performance across all categories, we additionally report the mean Intersection over Union (mIoU):

mIoU: The arithmetic mean of IoU values for all classes, which can comprehensively reflect the balanced segmentation accuracy of the model across different classes.


$$\text{mIoU}=\frac{1}{N_{c}}\sum_{c=1}^{N_{c}}{\text{IoU}}_{c}$$

Where $N_{c}$ is the total number of classes, and ${\text{IoU}}_{c}$ is the Intersection over Union of class c.

Through the optimization of network structure and training strategies, the improved model exhibited distinct trends in training loss, validation loss and all evaluation metrics over 150 epochs. It enhanced segmentation accuracy and robustness while maintaining high recall, and demonstrated outstanding performance especially in small target edge segmentation and adaptability to complex environments. This lays a more reliable technical foundation for subsequent agricultural applications such as high-precision seedling growth analysis and pest and disease detection.

### 3.3 Comparative experiments

This study focuses on the core scheme of improving the PointNet++ semantic segmentation model combined with the optimal clustering algorithm to achieve instance segmentation of eggplant seedling point clouds. Three groups of comparative experiments were designed to sequentially verify the gain effect of background purification preprocessing, the performance advantages of the improved semantic segmentation model, and the adaptability of the optimal clustering algorithm. The three parts proceed progressively and jointly support the selection and construction of the optimal instance segmentation scheme.

The semantic segmentation module is responsible for organ-level labeling of point clouds (stem, leaf, and background), providing fine-grained semantic information for downstream tasks. The instance segmentation module (based on the DBSCAN clustering algorithm) (as shown in **Figure 5E**), building upon the results of semantic segmentation, performs cluster partitioning on point clouds that belong to the same semantic category but originate from different individual seedlings, thereby achieving accurate separation at the single-seedling level. Each component supports the others with clear division of labor, collectively ensuring the effectiveness of the instance segmentation scheme.

All experiments strictly followed the control variable principle, with unified experimental datasets, evaluation metrics, and hardware environments to avoid interference from irrelevant factors. Meanwhile, targeted regulation strategies were formulated for issues such as data deviation and model overfitting during the experiments, ensuring the reliability and comparability of experimental results.

Through the three groups of comparative experiments, the optimal schemes for data preprocessing, semantic segmentation model, and clustering algorithm were determined respectively. These three components were then organically integrated, ultimately forming a complete technical solution for instance segmentation of eggplant seedling point clouds with both high precision and high efficiency.

#### 3.3.1 Comparison before and after background purification

In this experiment, model performance was directly compared under two input conditions: “retaining the seedling tray background” and “retaining only seedling stems and leaves”. For the first time in the semantic segmentation of eggplant seedling point clouds, the gain effect of background purification was quantitatively verified. 

**Table 4:** Segmentation performance comparison parameters of PointNet++ model before and after background purification.

Model	Precision	Recall	F1	Iou
Pointnet++(without background purification)	82.67%	85.29%	90.42%	77.96%
Pointnet++(background purification)	87.4%	89.95%	93.32%	76.97%

As shown in **Table 4** after background purification, the model’s Precision increased by 4.73%, Recall by 4.66%, and F1-score by 2.90%, indicating that removing the seedling tray background points helps reduce background misclassification and foreground omission. The IoU decreased from 77.96% to 76.97%, a reduction of 0.99%. This decrease is attributed to the removal of some edge points located at the boundary between the foreground and background during the background purification process. A paired t-test based on five repeated experiments showed that the IoU difference before and after background purification was not statistically significant (p > 0.05), indicating that the decline is not statistically significant and that background purification did not have a substantial adverse effect on IoU. Overall, background purification improves the model’s ability to identify foreground targets while maintaining segmentation accuracy. In terms of efficiency, background purification reduces the amount of input data, shortening the model’s forward inference time by approximately 20%–25% and reducing memory usage by approximately 18%.

#### 3.3.2 Comparison of semantic segmentation models

The core subjects of this comparative experiment are the original PointNet++ model and the proposed EggplantPointNet++ model in this study, and quantitative indicators are adopted to directly demonstrate the performance improvement of eggplant semantic segmentation brought by the proposed improvement strategies. The experiment focuses on comparing the feature extraction capability, semantic annotation accuracy and inference efficiency of each model, so as to verify the effectiveness of the improvement strategies. Models including DGCNN, PointCNN, PlantPointNet, DRP-Net, MS-PCNet and FPN-Point are not included in the core comparison system in this study, and the reasons are as follows: As general point cloud segmentation models, DGCNN and PointCNN have no architectural design tailored to the characteristics of agricultural seedling point clouds, such as small scale, heavy occlusion and class imbalance, so direct transfer to the research scenario in this paper will lead to significant segmentation adaptability problems, making it difficult to objectively verify the pertinence of the improvement strategies proposed in this study (Fareed et al., 2023). Although PlantPointNet is a dedicated agricultural model, it only introduces a single attention mechanism improvement based on the original PointNet, and its basic architecture is essentially different from that of PointNet++; including it in the comparison will make the results unobjective due to discrepancies in network hierarchy and feature learning logic.

Despite focusing on agricultural small-target segmentation, DRP-Net and MS-PCNet are quite different from the core improvement idea of “background purification + collaborative optimization of multiple modules” in this paper: the former prioritizes adaptability across different growth stages, while the latter lacks global context modeling, making it hard to accurately separate the gain effect of a single improved module. FPN-Point is excluded because its parameter volume and computational complexity are far higher than those of the model in this study, and the comparison will introduce additional variables due to differences in hardware resource occupation, failing to fairly verify the effectiveness of the lightweight improvement strategies. Therefore, the original PointNet++ with the same architecture is selected as the core comparison benchmark in this study, ensuring that the only experimental variable is the improved modules proposed in this paper, so as to accurately and objectively verify the actual gain effects of the improvement strategies including multi-scale residual blocks, channel attention and global context modules.

**Figure 6:**
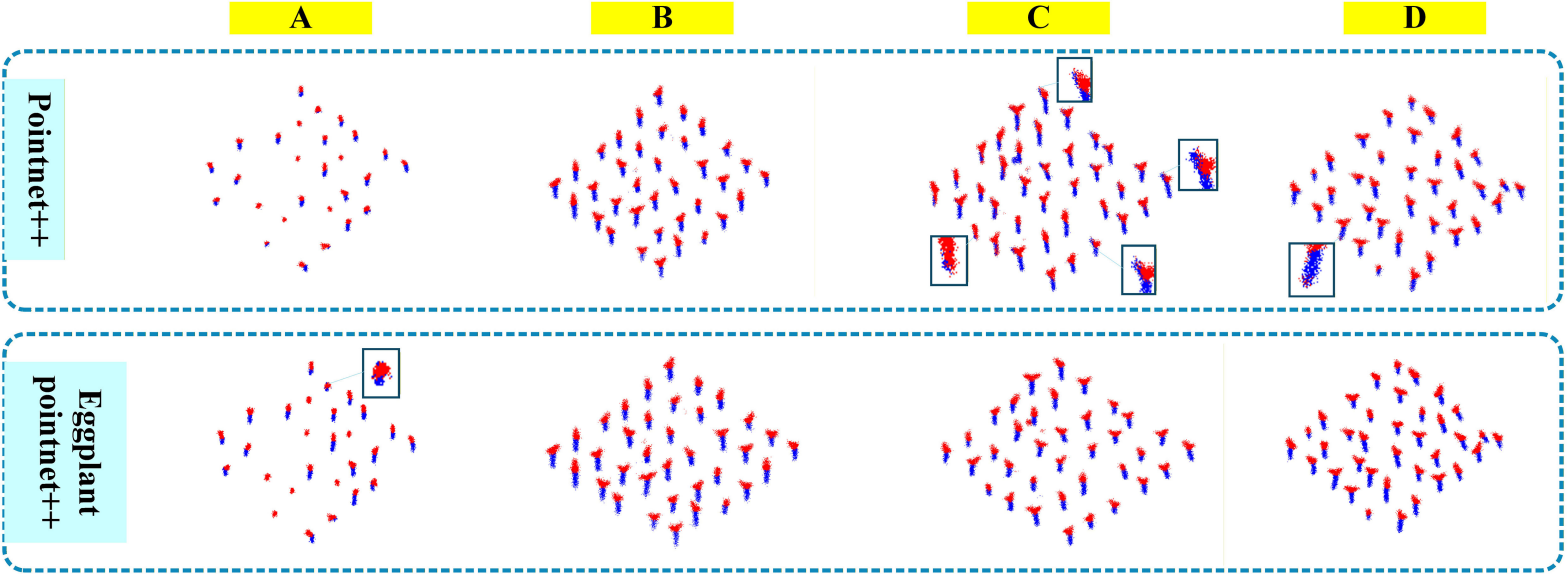
Comparison of semantic segmentation before and after improvement. Segmentation errors are highlighted.

The core performance parameters of semantic segmentation for the original PointNet++ and EggplantPointNet++ are compared in **Table 5**, and the visualization results are shown in **Figure 6**. To ensure the objectivity and reliability of the comparison results, the experiment adopts identical training configurations (unified learning rate, iteration times, batch size and other parameters), the same eggplant point cloud dataset and the same preprocessing process. The core quantitative indicators include Accuracy and mean Intersection over Union (mIoU), which are used to evaluate the semantic annotation accuracy and training stability of the models.

**Table 5:** Comparative analysis of semantic segmentation performance.

Indicators	Pointnet++	Eggplantpointnet++
Overall accuracy	87.40%	95.49%
Average Iou	76.97%	91.02%
Stem Iou	72.90%	89.24%
Leaf Iou	81.00%	92.79%
Stem accuracy	85.00%	93.27%
Leaf accuracy	90.00%	96.97%

As shown in **Table 5**, the improved PointNet++ outperforms the original model across all metrics. The overall accuracy increases from 87.40% to 95.49%, and the mean IoU rises from 76.97% to 91.02%, indicating that the improved model has a clear advantage in overall segmentation accuracy.

At the organ level, the improvement in stem IoU (16.34 percentage points) is greater than that in leaf IoU (11.79 percentage points). Similarly, the increase in stem classification accuracy (8.27 percentage points) exceeds that of leaf classification accuracy (6.97 percentage points). This discrepancy may be attributed to the stem being a slender structure with fewer points in the raw point cloud and being more prone to confusion with the background. The improved model enhances the ability to capture local slender geometric features through the multi-scale residual blocks, leading to greater improvements in stem segmentation accuracy.

Furthermore, the improved model maintains good consistency between IoU and classification accuracy for both stems and leaves, with both types of metrics improving simultaneously. This indicates that the model’s ability to fit organ boundaries and its discriminative power for category classification have both been enhanced, rather than only one aspect being optimized. Collectively, these results demonstrate that EggplantPointNet++ achieves substantial improvements in both overall segmentation accuracy and fine-grained recognition of stem and leaf organs, providing a more reliable semantic basis for subsequent instance segmentation of individual seedlings.

#### 3.3.3 Comparison of clustering algorithms

The comparison of clustering algorithms focuses on the adaptability to semantic segmentation results and the final performance of instance segmentation. Mainstream clustering algorithms suitable for point cloud data were selected, with the eggplant point cloud semantic feature maps and category annotation results output by the EggplantPointNet++ model as the unified input. We compared the clustering accuracy, rationality of cluster partitioning and corresponding instance segmentation performance of each algorithm, and screened out the clustering algorithm that is the most adaptable to the improved semantic segmentation results and can achieve the optimal eggplant instance segmentation effect, thus providing support for the determination of the final instance segmentation scheme (Zhao et al., 2024). For this comparison of clustering algorithms and evaluation of instance segmentation performance, Precision, Recall, F1 Score and mean Intersection over Union (mIoU) were adopted as the core quantitative metrics to comprehensively evaluate the cluster partitioning capability of each algorithm for eggplant targets and the accuracy of instance segmentation.

The selected comparison algorithms cover different clustering principles and include both classic algorithms and special-purpose algorithms adapted for point cloud data, specifically five algorithms: K-Medoids, K-Means, DFSP, Euclidean Clustering, and DBSCAN. Experimental variables were controlled uniformly: all algorithms adopted the same input data (semantic features output by EggplantPointNet++), and their core parameters were optimized via grid search (e.g., the K value for K-Means and K-Medoids, the neighborhood radius and minimum number of samples for DBSCAN, the distance threshold for Euclidean Clustering, etc.). This ensured that all algorithms participated in the comparison with optimal parameters, avoiding the impact of parameter setting deviations on the objectivity of experimental conclusions and instance segmentation performance evaluation (Ester et al., 1996).

**Figure 7:**
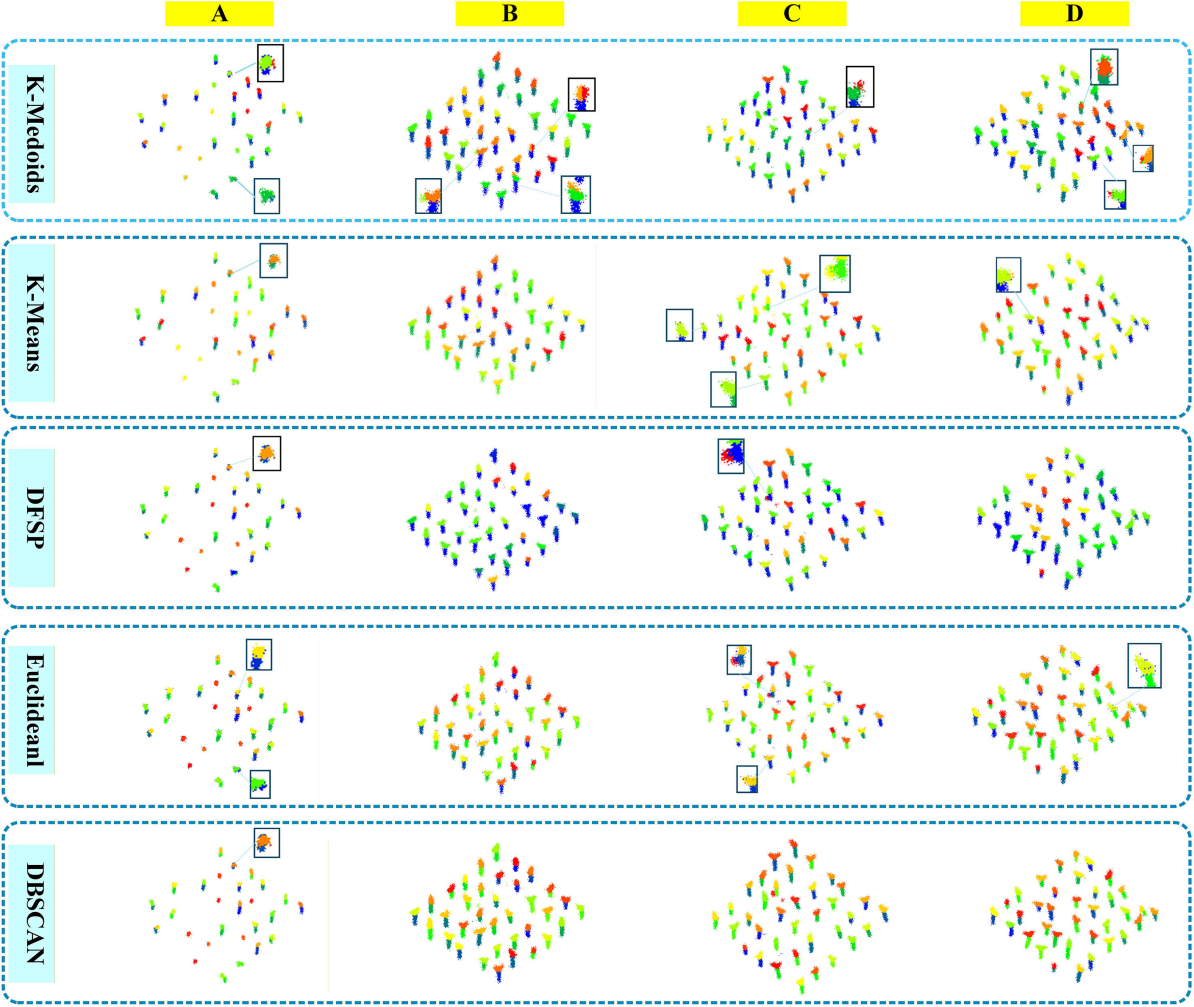
Comparison of clustering algorithm results.

The comparison results of various clustering algorithms are shown in **Table 6**, and the visualization results are presented in **Figure 7**.

**Table 6:** Comparison of core performance parameters for eggplant seedling instance segmentation with different clustering algorithms.

Eggplantpointnet++(+)	K-Medoids	K-means	DFSP	Euclidean clustering	DBSCAN
Precision	0.54	0.75	0.87	0.98	0.98
Recall	0.19	0.37	0.43	0.93	0.95
F1-Score	0.28	0.49	0.55	0.96	0.97
mIou	0.73	0.69	0.91	0.99	0.99

As shown in **Table 6**, the instance segmentation performance varied significantly across different clustering algorithms. DBSCAN and Euclidean Clustering achieved the highest levels of Precision, Recall, F1-Score, and mIoU. Specifically, DBSCAN achieved a Precision of 0.98, equal to that of Euclidean Clustering; a Recall of 0.95, which is 0.02 higher than that of Euclidean Clustering (0.93); an F1-Score of 0.97, 0.01 higher than that of Euclidean Clustering (0.96); and an mIoU of 0.99 for both algorithms. These results indicate that both algorithms enable high-precision cluster partitioning, with DBSCAN exhibiting a slight advantage over Euclidean Clustering in terms of completeness of target detection (Recall) and the trade-off between precision and recall (F1-Score).

DFSP achieved a Precision of 0.87, Recall of 0.43, F1-Score of 0.55, and mIoU of 0.91, all substantially lower than those of the top two algorithms. Its notably low Recall indicates a high rate of missed detections and insufficient coverage of seedling targets.

K-Medoids and K-Means exhibited the poorest performance. K-Medoids achieved a Precision of 0.54, Recall of 0.19, F1-Score of 0.28, and mIoU of 0.73; K-Means achieved a Precision of 0.75, Recall of 0.37, F1-Score of 0.49, and mIoU of 0.69. The metrics of both algorithms were significantly lower than those of the other algorithms, rendering them inadequate for meeting the application requirements of instance segmentation.

The training configuration information of DBSCAN is shown in **Table 7**.

**Table 7:** DBSCAN basic parameter configuration matrix.

Parameter	Select by category	Stem	Leaf
eps	eps = α × eps_est eps_est	eps_est × 0.8	eps_est × 1.2
min_samples	k + 1 = 5	5	5
metric	‘euclidean’	Euclidean distance	Euclidean distance
algorithm	Select by category	‘ball_tree’	‘kd_tree’
leaf_size	fixed value	30	30
n_jobs	-1	All CPU cores	All CPU cores

### 3.4 Ablation experiments

To investigate the contribution of each improved module to the final performance, ablation experiments were conducted in this study. Comparative training was performed by sequentially removing or replacing the key components in the model, and the results are shown in the table.

The multi-scale convolution module extracts multi-scale features of plant point clouds synchronously through a parallel multi-branch structure with 1×1, 3×3 and 5×5 convolution kernels, which effectively captures hierarchical information ranging from leaf textures to the overall plant structure. The channel attention module adopts a dual-path pooling aggregation mechanism to dynamically recalibrate the importance of feature channels, enhancing key features and suppressing noise. The global context module models long-range dependency relationships through a squeeze-and-excitation framework, enabling the understanding of topological connections between plant organs.

As shown in **Table 8**, each improved module contributed to varying degrees of performance enhancement. When the multi-scale convolution module (MS) was introduced alone, mIoU increased from 85.72% to 88.90%, representing an improvement of 3.18 percentage points. The channel attention module (CA) alone raised mIoU to 87.16%, an increase of 1.44 percentage points. The global context module (GC) alone resulted in an mIoU equal to that of the baseline (85.72%).

**Table 8:** Analysis of ablation experiment results.

configuration	Total parameters	Relative increment	calculateFLOPs(G)	Accuracy(%)	mIoU(%)
Baseline	0.97	–	1.2	92.47%	85.72%
+MS	5.35	+451%	6.8	94.24%	88.90%
+SE Block	1.03	+6%	1.4	93.24%	87.16%
+GC	1.01	+0.4%	1.3	92.47%	85.72%
+MS+SE Block	5.42	+459%	7.1	93.39%	87.43%
+MS+GC	5.40	+457%	7.0	93.89%	88.27%
+GC+SE Block	1.08	+11%	1.5	93.27%	87.22%
Full_model	5.46	+463%	7.3	95.49%	91.02%

Among the pairwise combinations, the MS+GC configuration achieved an mIoU of 88.27%, MS+CA reached 87.43%, and GC+CA attained 87.22%, all outperforming any single-module configuration. Although the mIoU of the MS+GC combination (88.27%) was slightly lower than that of the MS module alone (88.90%), the difference in accuracy (from 94.24% to 93.89%) was marginal.

The full model, incorporating all three modules, achieved the best performance among all configurations, with an mIoU of 91.02% and an accuracy of 95.49%. Compared with the baseline, the full model exhibited an mIoU improvement of 5.30 percentage points; relative to the best single-module configuration (MS alone), the improvement was 2.12 percentage points. In terms of computational complexity, the full model has 5.46M parameters and 7.3G FLOPs, which, while higher than those of the baseline (0.97M, 1.2G), remain within an acceptable range. Overall, the combination of all three modules yielded the greatest performance gain, indicating that the multi-scale convolution module, channel attention module, and global context module are highly complementary.

## 4 Calculation of phenotypic parameters of eggplant seedlings

### 4.1 Size restoration and physical scale recovery

Before extracting individual seedling point clouds, it is necessary to restore the point cloud data to their true physical dimensions to ensure the accuracy of subsequent phenotypic parameter calculations. The size restoration in this study is based on a dual mechanism.

First, the scale calibration factor k established in Section 2.3.2 (derived from the known dimensions of the cultivation tray) is applied to uniformly scale the spatial coordinates of the point cloud, converting the virtual scale in the reconstructed coordinate system to actual physical dimensions.

Second, because random scaling operations were applied to training set samples during data augmentation, the scaling factor for each sample has been meticulously recorded, and an association of “sample ID – augmentation type – parameter value” has been established. During the phenotypic parameter extraction phase, the corresponding scaling factor is traced based on the current sample ID, and the point cloud dimensions are restored to their original physical state prior to data augmentation.

This dual size restoration mechanism effectively eliminates dimensional deviations introduced by three-dimensional reconstruction errors and data augmentation, ensuring that the calculated results of phenotypic parameters such as plant height, stem diameter, and cotyledon area have a traceable physical benchmark and reproducibility. All subsequent phenotypic parameter calculations are performed on the size-restored point cloud data.

### 4.2 Single-plant extraction

**Figure 8:**
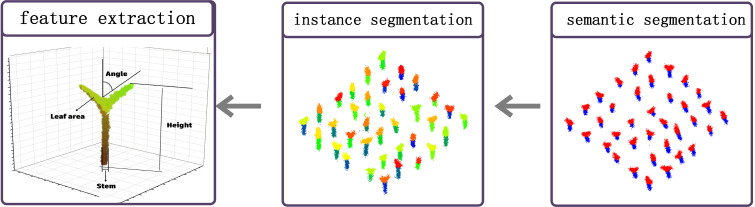
Schematic diagram of single seedling extraction.

Based on the instance segmentation scheme combining the EggplantPointNet++ semantic segmentation model and the optimal clustering algorithm, this study completed the accurate segmentation of eggplant seedling point clouds, extracted the point cloud data of individual eggplant seedlings, (as shown in **Figure 8**) and provided high-quality data support for the calculation of phenotypic parameters. Focusing on the key indicators of eggplant seedling growth, the phenotypic analysis selected four core phenotypic parameters, namely plant height, stem diameter, leaf angle and surface area. Through quantitative analysis, the accurate evaluation of the growth state of eggplant seedlings was realized, which solved the problems of low efficiency, easy damage to seedlings and large errors in traditional manual measurement, and achieved non-destructive, efficient and accurate detection of eggplant seedling phenotypes. 

### 4.3 Calculation and result analysis of core phenotypic parameters

#### 4.3.1 Plant height calculation

The plant height of eggplant seedlings is defined as the vertical distance from the contact point of the seedling with the soil to the top of the seedling. In this study, the plant heights of four groups of eggplant seedlings were calculated, which clearly showed the growth law of eggplant seedlings with time (Madec et al., 2017). All data points of the point cloud of a single eggplant seedling were traversed, and the maximum and minimum values of the Z-axis coordinates were extracted. The difference between the two values is the plant height, and the formula is as follows:


$$\text{H}={\text{z}}_{\text{max}}-{\text{z}}_{\text{min}}$$

Where, H is the plant height of eggplant seedlings, ${\text{z}}_{\text{max}}$ is the maximum value of the Z-axis coordinates of the point cloud, and ${\text{z}}_{\text{min}}$ is the minimum value of the Z-axis coordinates of the point cloud (Zhang et al., 2024).

**Figure 9:**
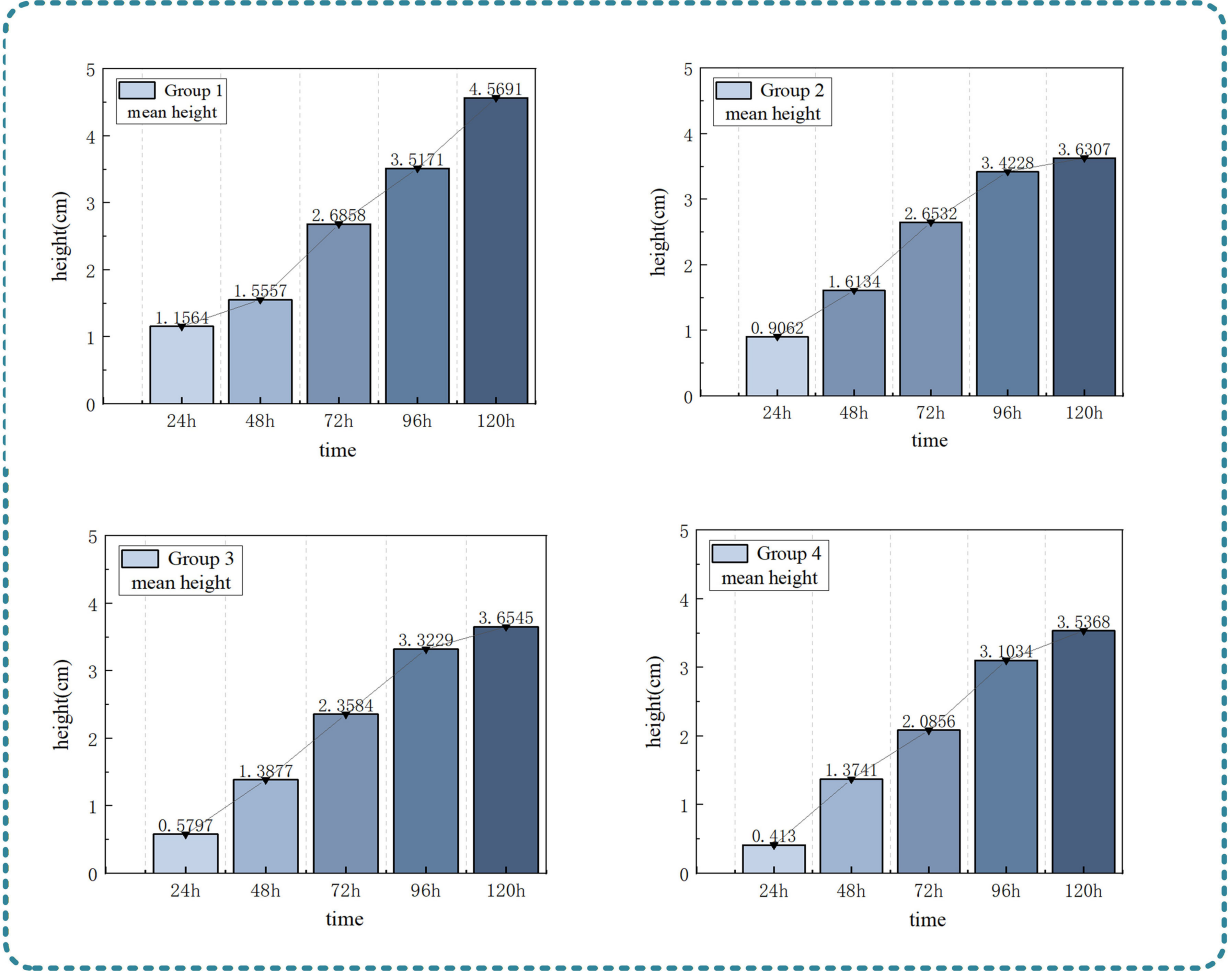
Bar chart of plant height variation.

Result analysis: (as shown in **Figure 9**) At the initial stage of cultivation (24h), the seedlings had just emerged from the soil, and the cotyledons were not unfolded. The average plant height of the four boxes of seedlings was generally lower than 1.5cm, and the heights of the seedling population were similar, indicating that the growth of the seedlings was slow during the emergence stage after seed germination. When cultured to 48–72h, the cotyledons gradually unfolded and grew upward, the growth rate of the average plant height increased significantly, the overall height of the seedling population in the visual images increased obviously, and the advantage of the average plant height of seedlings in some boxes was initially highlighted. At 72h, the average plant height of the four boxes of seedlings increased by 67.3% compared with that at 24h. At 120h, the cotyledons basically unfolded to the maximum extent, and the average plant height of the four boxes of seedlings reached the peak at the cotyledon stage. The height difference of the four boxes of seedling populations in the visual images was clearly visible, and the difference in average plant height was stably around 0.3cm. This further confirms the consistency with the growth and physiological characteristics of seedlings at the cotyledon stage, and also verifies the accurate monitoring ability of 3D point cloud technology for plant height indicators at the stage without true leaves, providing reliable data support and intuitive visual basis for the grading of seedling growth potential at the cotyledon stage. 

#### 4.3.2 Stem diameter

Stem diameter is a core phenotypic parameter reflecting the growth potential of eggplant seedlings during the cotyledon stage, which is directly related to the water transport capacity and nutrient supply efficiency of seedlings, and also affects the stress resistance of seedlings. It is defined as the average diameter of the stem of eggplant seedlings 0.5cm above the ground surface, avoiding the interference of surface soil occlusion and weak top parts. In this study, the stem diameters of four groups of eggplant seedlings were calculated, which clearly showed the change law of the stem diameter of eggplant seedlings with time. The calculation process is as follows: Based on the aligned point cloud data, the point clouds with Z-axis coordinates in the range of [z_min + 4.5cm, z_min + 5.5cm] were selected as the target stem region (z_min is the minimum Z-axis coordinate of the seedling point cloud, i.e., the coordinate of the contact point with the soil); The selected stem point cloud was denoised to eliminate discrete noise points, and the minimum enclosing circle fitting algorithm (Gander et al., 1994) was used to perform circular fitting on the stem point cloud to obtain the radius of the enclosing circle; Finally, the diameter was calculated by the radius, and the fitting was repeated 5 times to take the average value as the final stem diameter to reduce the fitting error. The formula is as follows:


$$\text{D}=2\times \bar{\text{r}}$$

Where, D is the stem diameter of eggplant seedlings, and **r** is the average value of the radii obtained by 5 times of minimum enclosing circle fitting. This method can accurately capture the cross-sectional morphology of the seedling stem, adapt to the characteristics of thin and irregular shape of the eggplant seedling stem, and realize non-destructive and accurate quantification of stem diameter.

**Figure 10:**
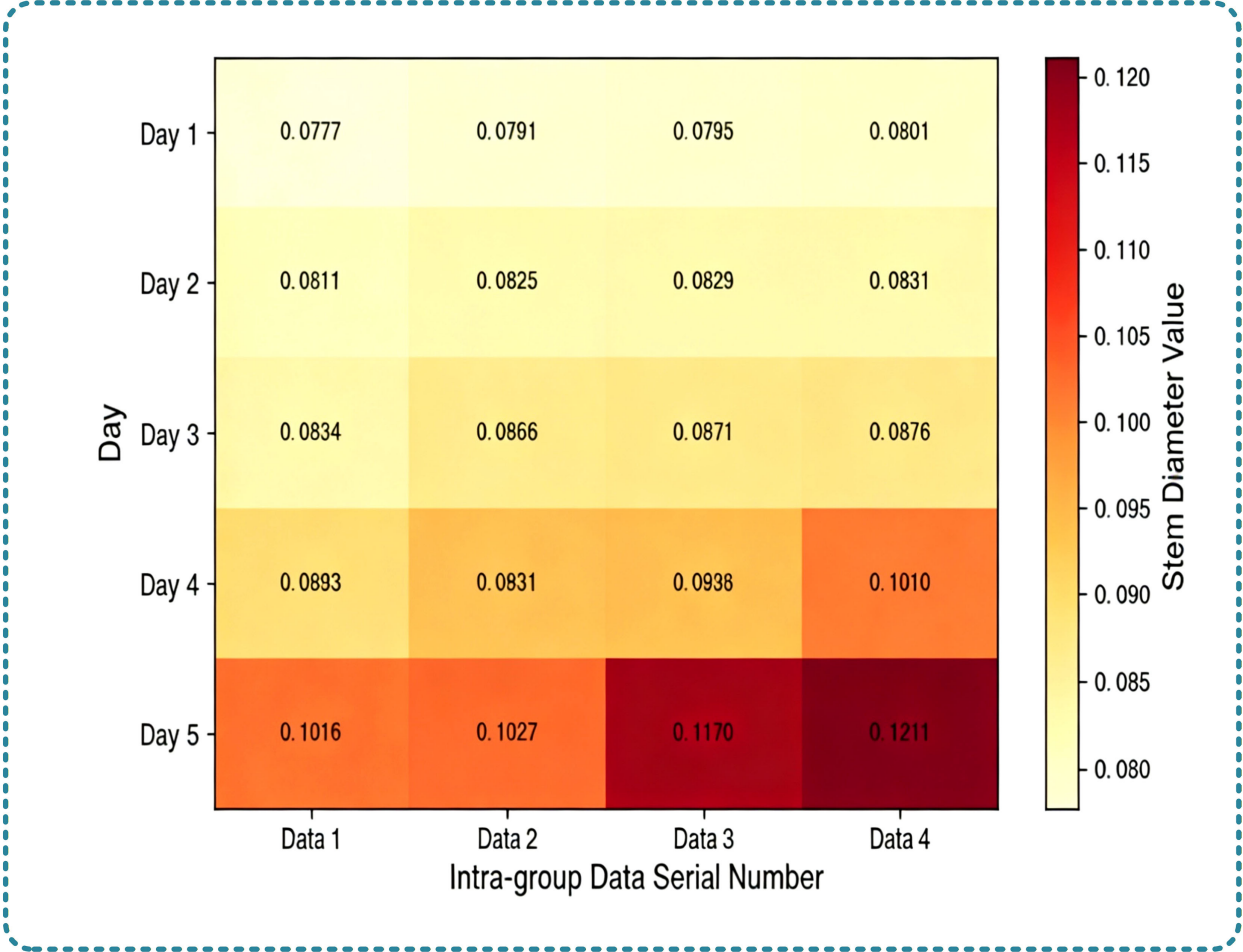
Heat map of stem diameter variation.

Result analysis: In the first 24h of cultivation, the stem diameter of seedlings was generally in the range of 0.06–0.08cm, and the color distribution of the heat map was uniform without obvious depth difference as shown in **Figure 10**. This indicates that the stem was in the cell differentiation and accumulation stage at the initial cotyledon stage, with slow thickening. At this time, the water transport capacity of seedlings was weak, which could only meet the basic water demand under the closed cotyledon state. After 48h, with the increase of water and nutrient demand for cotyledon expansion, the growth rate of stem diameter accelerated. At 72h, the average stem diameter reached 0.102cm, an increase of 38.5% compared with 24h. In the heat map, the color of the dominant seedling area was significantly deepened, indicating that such seedlings had thicker stems and stronger water transport capacity, which could provide more sufficient water support for cotyledon expansion. At 120h, the stem diameter reached the maximum value at the cotyledon stage, and the color stratification of the heat map was more distinct. The stem diameter differences among the 6 seedlings were stably presented. This result proves that the stem diameter calculation method based on the minimum circumscribed circle fitting can accurately capture the subtle growth changes of the seedling stems at the cotyledon stage. Meanwhile, combined with the heat map, the differences in water transport capacity of seedlings can be intuitively presented, providing a key basis for evaluating the water transport efficiency of seedlings at the cotyledon stage and screening dominant seedlings. 

#### 4.3.3 Inclination angle calculation

Cotyledon inclination angle is a key parameter reflecting the photosynthetic posture of eggplant seedlings during the cotyledon stage, which is defined as the angle between the cotyledon plane and the vertical plane (the average value of the inclination angles of the two cotyledons is taken as the final inclination angle of a single seedling). This parameter directly affects the capture efficiency of cotyledons for photosynthetically active radiation. The increase of inclination angle with growth time can enable cotyledons to receive more sufficient light and improve photosynthetic efficiency. The calculation process is as follows: First, based on the segmented eggplant seedling point cloud, the cotyledon area point cloud was selected to eliminate stem interference; The Random Sample Consensus (RANSAC) algorithm was used to perform plane fitting on the cotyledon point cloud to obtain the normal vector $\vec{\text{n}}$ =(a,b,c) of the cotyledon plane; Finally, the cotyledon inclination angle was solved according to the angle between the normal vector of the cotyledon plane and the normal vector $\vec{\text{z}}$ =(0,0,1) of the vertical plane (i.e., the Z-axis direction perpendicular to the tray plane and along the gravity direction). The formula is as follows:


$$\text{θ}=\arccos(\frac{|\vec{\text{n}}\cdot \vec{\text{z}}|}{|\vec{\text{n}}|\cdot |\vec{\text{z}}|})\times \frac{180^{\circ }}{\text{π}}$$

Where, θ is the cotyledon inclination angle of eggplant seedlings (unit: °), $\vec{\text{n}}\cdot \vec{\text{z}}$ is the dot product of the two vectors, and $|\vec{\text{n}}|$and, $|\vec{\text{z}}|$are the moduli of the two vectors respectively. For a single seedling, the average value of the inclination angles obtained by fitting the two cotyledons was taken, and the calculation was repeated 3 times to reduce the plane fitting error and ensure the accuracy of the inclination angle calculation (Cai et al., 2021).

**Figure 11:**
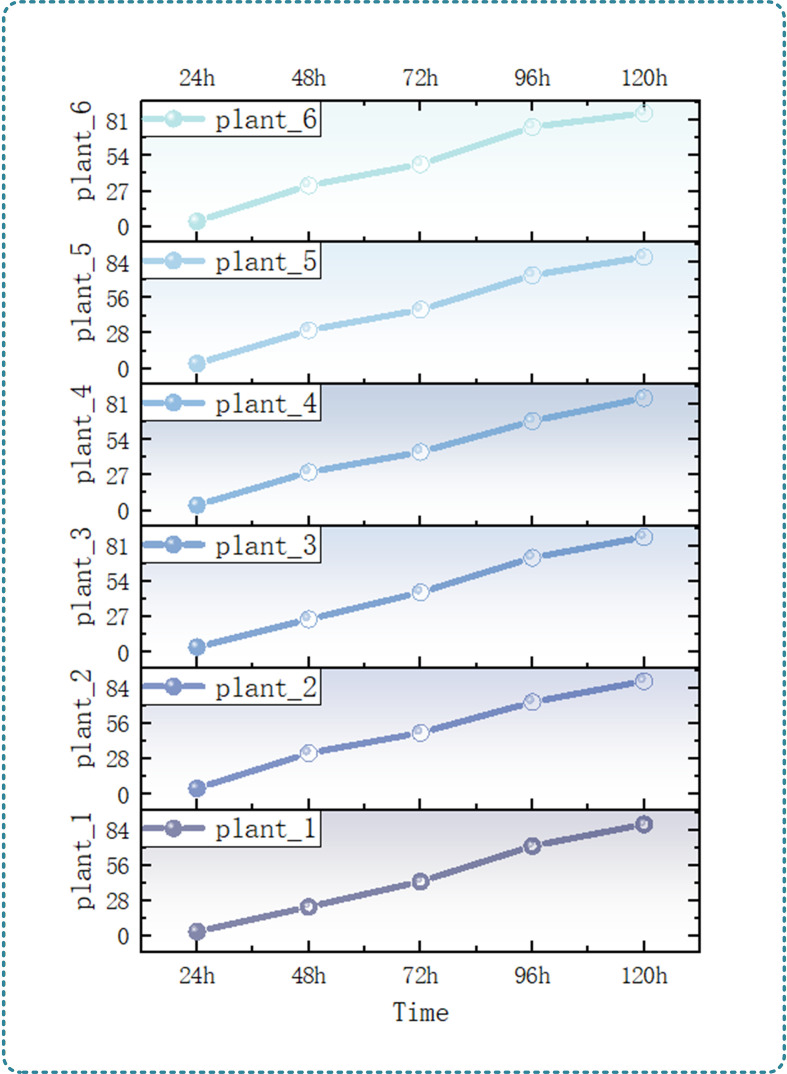
Leaf angle variation of a single seedling.

Result analysis: Combined with the temporal monitoring results of cotyledon angle as shown in **Figure 11**, it can be seen that the cotyledon angle of eggplant seedlings showed a continuous increasing trend with the extension of culture time at the cotyledon stage, which was highly consistent with the cotyledon expansion process and photosynthesis demand, and the angle change was directly related to the cotyledon expansion state. At the initial stage of cultivation (24h-48h), the cotyledons had just emerged from the soil and were in a closed state, and the angle with the vertical plane was generally small (0°-30°), at which time the capture area of photosynthetically active radiation was limited. With the cultivation time advancing to 72h, the angle between cotyledons and the vertical plane increased significantly, and the average angle reached 30°-50°, forming a better angle with the light direction. At 120h, the cotyledons were basically fully expanded, and the angle with the vertical plane reached the peak at the cotyledon stage (50°-90°) and tended to be stable. At this time, the cotyledons were closest to the horizontal state, which could receive light more fully, maximize the capture efficiency of photosynthetically active radiation, and provide sufficient photosynthates for seedling growth. This result verifies the accurate quantification ability of 3D point cloud technology combined with plane fitting algorithm for the spatial attitude of cotyledons at the cotyledon stage, providing key parameter support for evaluating the photosynthetic potential and growth vitality of seedlings at the cotyledon stage. 

#### 4.3.4 Cotyledon area calculation

In this study, the surface area refers to the cotyledon area, which is the most core phenotypic parameter of eggplant seedlings during the cotyledon stage. It directly determines the capture amount of photosynthetically active radiation, thereby affecting the smooth progress of photosynthesis and the accumulation of photosynthates, and ultimately regulating the growth rate and growth potential of seedlings. Six individual cotyledons were randomly selected for continuous morphological tracking in the experiment. By extracting their leaf area characteristic parameters and combining with the corresponding tracking images, the growth law of cotyledon area and its influence on seedling photosynthesis were systematically analyzed.

The Ball Pivoting Algorithm (BPA) was used to estimate the surface area of eggplant seedlings. This algorithm simulates the rolling of a virtual ball on the surface of the point cloud, captures three-point contacts to form triangular meshes, and then obtains the total surface area by summing the areas of the triangles, which can accurately reflect the photosynthetic area and resource utilization capacity of seedlings (Bernardini et al., 1999)The radius of the virtual ball was set to 0.2cm. After generating a complete triangular mesh, the area of each triangle was calculated by Heron’s formula, and the sum was taken to obtain the total surface area of eggplant seedlings. The formula is as follows:


$$\text{A}=\sum_{\text{j}=1}^{\text{k}}{\text{S}}_{\text{j}}$$

Where, A is the total surface area of eggplant seedlings, k is the total number of triangular meshes, and ${\text{S}}_{\text{j}}$ is the area of the j-th triangle (Zabawa et al., 2021).

**Figure 12:**
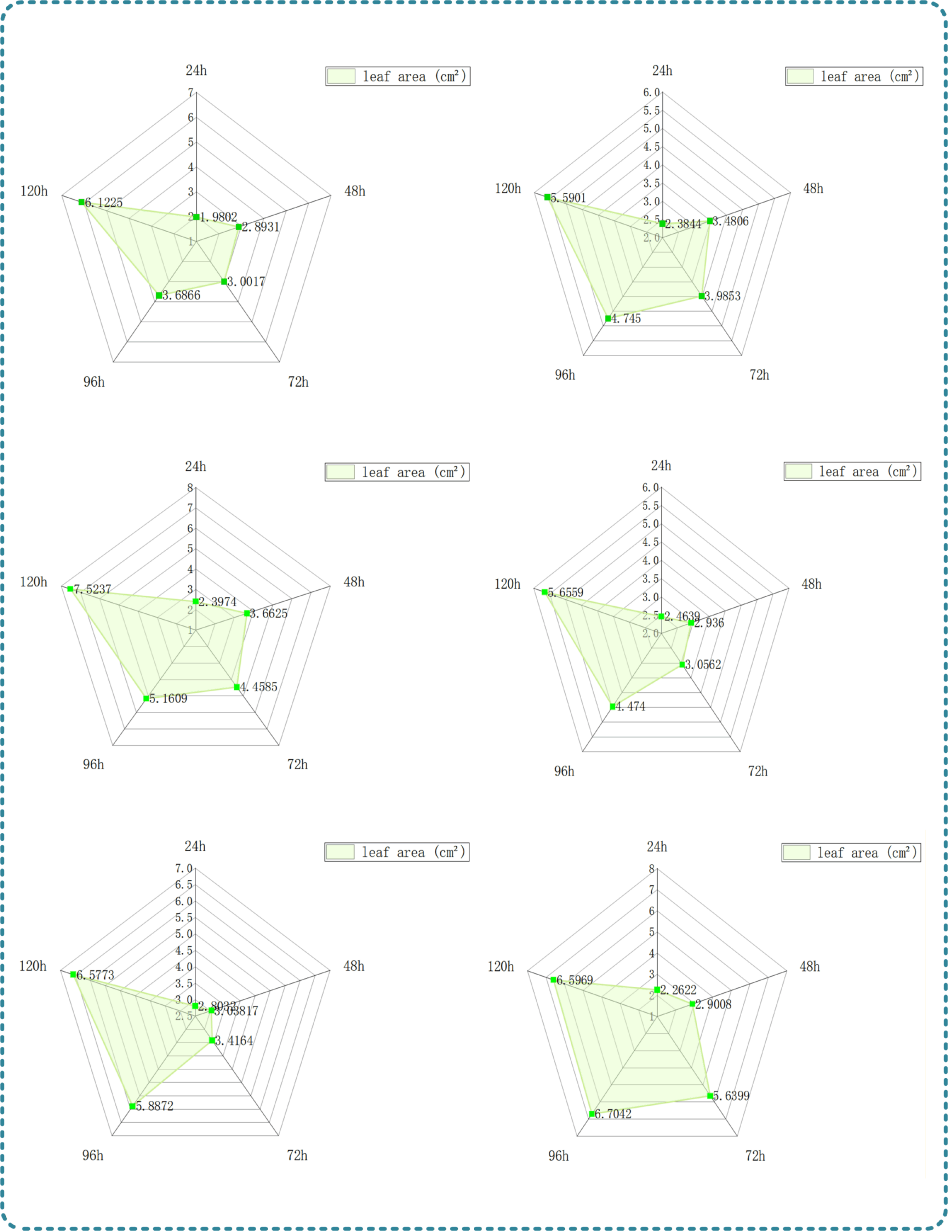
Cotyledon area variation of a single seedling.

Result analysis: Combined with the randomly selected tracking images of 6 individual cotyledons and the extracted leaf area characteristic parameters as shown in **Figure 12**, it can be seen that in the first 24h of cultivation, the cotyledons were in a closed state, and the leaf area was generally less than 0.8cm². At this time, the photosynthetic area of the cotyledons was limited, and the photosynthesis intensity was weak, which could only accumulate a small amount of photosynthates to meet their own basic metabolism. The period of 48–72h was the key stage for cotyledon expansion and leaf area growth, during which the leaf area growth rate reached the maximum. It can be seen from the tracking images that the cotyledons gradually unfolded and flattened, the capture area of photosynthetically active radiation was greatly increased, and the photosynthesis efficiency was significantly enhanced, providing sufficient energy support for the growth of seedling plant height and stem diameter. At 120h, all 6 cotyledons were basically fully expanded, and the leaf area reached the peak at the cotyledon stage. At this time, the photosynthetic area of the cotyledons was the largest, and the photosynthesis intensity tended to be stable. Further analysis of the individual differences among the 6 cotyledons showed that the larger the leaf area, the better the unfolding degree of the cotyledons, and the better the corresponding seedling growth potential, which confirms the decisive impact of leaf area on photosynthesis — the larger the leaf area, the more photosynthetically active radiation captured, the more sufficient the photosynthesis, the more photosynthates accumulated, and the better the seedling growth state. In summary, combined with the tracking images and characteristic parameters of 6 individual cotyledons, it can be clearly concluded that leaf area is the most important phenotypic parameter of eggplant seedlings at the cotyledon stage, and its size and growth dynamics directly regulate the efficiency of photosynthesis, serving as a core indicator for evaluating the photosynthetic potential and growth state of seedlings. 

## 5 Conclusion

To achieve accurate and non-destructive quantification of phenotypic parameters of eggplant seedlings at the cotyledon stage, and to address the limitations of traditional two-dimensional phenotyping techniques—such as incomplete feature extraction, susceptibility to occlusion, and insufficient accuracy—as well as the challenges of existing three-dimensional phenotyping techniques, including strong background interference and large data volumes, this study employed seedlings of the variety ‘Jingnongyan Purple Red Long Eggplant’ as the research subject. We proposed an enhanced point cloud segmentation framework, EggplantPointNet++, and adopted point cloud background purification as a core preprocessing step, establishing a complete technical pipeline encompassing time-series data acquisition, three-dimensional point cloud reconstruction, background purification, precise segmentation, and phenotypic parameter calculation.

Background purification optimizes point cloud quality at the data source, providing a stable foundation for the technical pipeline. The EggplantPointNet++ model introduces multi-dimensional improvements to the original PointNet++ architecture by incorporating modules such as multi-scale residual blocks and channel attention mechanisms, thereby enhancing feature capture and modeling capabilities. The synergy between background purification and model improvement effectively improved segmentation performance. Compared with the original PointNet++ model without background purification, the optimized model achieved notable improvements in overall accuracy, mean intersection over union (mIoU), as well as stem and leaf IoU, providing a feasible solution for accurate segmentation and phenotypic quantification of eggplant seedlings.

In terms of data processing and parameter calculation, this study avoided the issue of missing features inherent in two-dimensional single-view imaging through time-series data acquisition. After obtaining high-purity target point clouds via background purification, data augmentation was applied exclusively to the training set, followed by dataset partitioning. The optimal DBSCAN clustering algorithm was selected to achieve accurate separation of individual seedling point clouds. Furthermore, we developed non-destructive quantification methods for five core phenotypic parameters, effectively overcoming the limitations of manual measurements. Background purification also ensured the reliability and accuracy of the entire technical pipeline.

Through a series of progressive comparative experiments, this study validated the effectiveness of the proposed technical pipeline. Background purification significantly improved model segmentation accuracy and operational efficiency, confirming the constraining effects of background interference and data redundancy on segmentation performance. The EggplantPointNet++ model demonstrated clear performance advantages over the original PointNet++. The DBSCAN clustering algorithm outperformed other comparative algorithms in the instance segmentation task. Validation of the phenotypic parameters showed that the calculated results were highly consistent with the physiological growth characteristics of eggplant seedlings, with temporal variation patterns matching the growth traits of seedlings at the cotyledon stage. This outcome was made possible by the high-purity point cloud data produced by the model.

The technical pipeline established in this study provides an automated method for accurate phenotypic monitoring of eggplant seedlings at the cotyledon stage. Background purification offers a transferable approach for addressing related issues in point cloud segmentation of similar crops. The lightweight potential of the EggplantPointNet++ model enhances the applicability of the technical pipeline. The developed phenotypic parameter quantification system can also serve as a reference for seedling growth assessment and superior seedling selection, thereby providing technical support for accurate phenotypic analysis of eggplant germplasm resources.

## Data Availability

The raw data supporting the conclusions of this article will be made available by the authors, without undue reservation.

## References

[B1] Bernardini F. Mittleman J. Rushmeier H. Silva C. Taubin G. (1999). The ball-pivoting algorithm for surface reconstruction. IEEE Trans. Visual Comput. Graphics 5, 349–359. doi: 10.1109/2945.817413. PMID: 25079929

[B2] Cai J. Liu J. Chen L. (2021). Direct and accurate feature extraction from 3D point clouds of plants using RANSAC. Comput. Electron. Agric. 187, 106240. doi: 10.1016/j.compag.2021.106240. PMID: 38826717

[B3] Chen H. Shi C. Li W. Duan C. Yan J. (2021). Multi-scale salient instance segmentation based on encoder-decoder. In Balasubramanian V. N. Tsang I. , eds. Proceedings of The 13th Asian Conference on Machine Learning (: PMLR) 157, 1445–1460. Available online at: https://proceedings.mlr.press/v157/chen21b.html (Accessed April 30, 2026).

[B4] Eelbode T. Maier-Hein K. Handels H. (2020). Revisiting evaluation metrics for semantic segmentation: Optimization and evaluation of fine-grained intersection over union. IEEE Trans. Med. Imaging 39, 3461–3472. doi: 10.1109/TMI.2020.3002417. PMID: 25079929

[B5] Ester M. Kriegel H. P. Sander J. Xu X. (1996). A density-based algorithm for discovering clusters in large spatial databases with noise. In Proceedings of the Second International Conference on Knowledge Discovery and Data Mining (KDD-96) (Portland, OR: AAAI Press), 226–231.

[B6] Everingham M. Van Gool L. Williams C. K. I. Winn J. Zisserman A. (2010). The pascal visual object classes (VOC) challenge. Int. J. Comput. Vision 88, 303–338. doi: 10.1007/s11263-009-0275-4. PMID: 30311153

[B7] Fang H. Zhu J. Hu P. Meng L. Zhu B. Guo Y. . (2018). Image-based dynamic quantification and high-accuracy 3D evaluation of canopy structure of plant populations. Ann. Bot. 121, 1079–1088. doi: 10.1093/aob/mcy016. PMID: 29509841 PMC5906925

[B8] Fareed N. Flores J. P. Das A. K. (2023). Analysis of UAS-LiDAR Ground Points Classification in Agricultural Fields Using Traditional Algorithms and PointCNN. Remote Sens. 15, 483. doi: 10.3390/rs15020483

[B9] Fischler M. A. Bolles R. C. (1981). Random sample consensus: A paradigm for model fitting with applications to image analysis and automated cartography. Commun. ACM 24, 381–395. doi: 10.1145/358669.358692

[B10] Gander W. Golub G. H. Strebel R. (1994). Least squares fitting of circles and ellipses. BIT Numer. Math. 34, 558–578. doi: 10.1007/BF01934190. PMID: 30311153

[B11] Goodfellow I. Bengio Y. Courville A. (2016). Deep learning (Cambridge, Massachusetts: The MIT Press). doi: 10.1017/CBO9781107415324.004

[B12] Grossnickle S. C. MacDonald J. E. (2018). Why seedlings grow: Influence of plant attributes. New For. 49, 1–34. doi: 10.1007/s11056-017-9606-4. PMID: 30311153

[B13] Han B. Li Y. Bie Z. Peng C. Huang Y. Xu S. Y. (2022). MIX-NET: deep learning-based point cloud processing method for segmentation and occlusion leaf restoration of seedlings. Plants 11, 3342. doi: 10.3390/plants11233342. PMID: 36501381 PMC9739940

[B14] Han B. Sun Z. Liu S. Cao Y. He J. Zhu Y. . (2025). A wheat posture monitoring and analysis system based on keypoint detection. Comput. Electron. Agric. 239, 110928. doi: 10.1016/j.compag.2025.110928. PMID: 38826717

[B15] Hu J. Shen L. Sun G. (2018). Squeeze-and-excitation networks. IEEE Trans. Pattern Anal. Mach. Intell. 42, 2011–2023. doi: 10.1109/TPAMI.2019.2913372. PMID: 31034408

[B16] Jin X. Tang L. Li R. Ji J. Liu J. (2022). Selective transplantation method of leafy vegetable seedlings based on ResNet 18 network. Front. Plant Sci. 13, 893357. doi: 10.3389/fpls.2022.893357. PMID: 35937327 PMC9355090

[B17] Kingma D. P. Ba J. (2015). Adam: A method for stochastic optimization. ICLR. doi: 10.48550/arXiv.1412.6980

[B18] Li Y. Bu R. Sun M. Wu W. Di X. Chen B. (2018). PointCNN: Convolution on X-transformed points. In Bengio S. Wallach H. M. Larochelle H. Grauman K. Cesa-Bianchi N. Garnett R. , eds. Advances in Neural Information Processing Systems 31: Annual Conference on Neural Information Processing Systems 2018, NeurIPS 2018, 3-8 December 2018, Montréal, Canada (Montreal, Canada: Curran Associates Inc.), 820–830.

[B19] Li X. Ouyang Z. Cheng Q. Zhong Z. Fu X. (2025). Investigation of salt stress effects on maize seedling phenotypic traits based on the Point CornNet point cloud segmentation model. Front. Plant Sci. 16, 1621509. doi: 10.3389/fpls.2025.1621509. PMID: 40995007 PMC12454380

[B20] Li J. Shao Y. Tian L. Zhang Z. Guo Y. Zhong Z. . (2026). PTV2-Fr: A point cloud segmentation network for phenotypic trait extraction and gibberellin effect analysis in sorghum seedlings. Front. Plant Sci. 17, 1761249. doi: 10.3389/fpls.2026.1761249. PMID: 41799987 PMC12961441

[B21] Li D. Zhang H. Chen W. Fu X. (2024). Lightweight multi-scale feature fusion for point cloud segmentation in resource-limited devices. J. Intell. Rob. Syst. 112, 47. doi: 10.1007/s10846-024-02315-x. PMID: 30311153

[B22] Li Y. Zhang X. Han S. Li G. (2022). PlantPointNet: A lightweight point cloud segmentation network for crop seedling phenotyping. Trans. Chin. Soc. Agric. Eng. 38, 165–173.

[B23] Lipton Z. C. Elkan C. Swamy B. N. (2014). Thresholding classifiers to maximize F1 score. arXiv preprint arXiv:1402.1892. (Ithaca, NY: Cornell University). doi: 10.48550/arXiv.1402.1892

[B24] Liu Z. Wang H. Chen L. (2024). DRP-Net: Dynamic receptive field propagation network for tomato and eggplant seedling stem-leaf segmentation. IEEE Trans. Instrum. Meas. 73, 1–12.

[B25] Long J. Shelhamer E. Darrell T. (2014). Fully convolutional networks for semantic segmentation. IEEE Trans. Pattern Anal. Mach. Intell. 39, 640–651. doi: 10.1109/TPAMI.2016.2572683. PMID: 27244717

[B26] Lyngdoh Y. A. Saha P. Tomar B. S. Bhardwaj R. Nandi L. L. Srivastava M. . (2025). Unveiling the nutraceutical potential of indigenous and exotic eggplant for bioactive compounds and antioxidant activity as well as its suitability to the nutraceutical industry. Front. Plant Sci. 16, 1451462. doi: 10.3389/fpls.2025.1451462. PMID: 39967813 PMC11832719

[B27] Lyu J. Jin N. Ma X. Yin X. Jin L. Wang S. . (2024). A comprehensive evaluation of nutritional quality and antioxidant capacity of different Chinese eggplant varieties based on multivariate statistical analysis. Antioxidants 14, 10. doi: 10.3390/antiox14010010. PMID: 39857344 PMC11761265

[B28] Madec S. Baret F. de Solan B. Thomas S. Dutartre D. Jezequel S. . (2017). High-throughput phenotyping of plant height: Comparing unmanned aerial vehicles and ground LiDAR estimates. Front. Plant Sci. 8, 2002. doi: 10.3389/fpls.2017.02002. PMID: 29230229 PMC5711830

[B29] Martinez-Guanter J. Ribeiro Á. Peteinatos G. G. Pérez-Ruiz M. Gerhards R. Bengochea-Guevara J. M. . (2019). Low-cost three-dimensional modeling of crop plants. Sensors 19, 2883. doi: 10.3390/s19132883. PMID: 31261757 PMC6651267

[B30] Omia E. Park E. Semyalo D. Joshi R. Cho B.-K. (2026). Advancements in 3D field-crop phenotyping using point clouds: A comparative review of sensor technology, target traits, and challenges under controlled and field conditions. Front. Plant Sci. 17, 1731852. doi: 10.3389/fpls.2026.1731852. PMID: 41727990 PMC12920483

[B31] Poorter H. Hummel G. M. Nagel K. A. Fiorani F. Von Gillhaussen P. Virnich O. . (2023). Pitfalls and potential of high-throughput plant phenotyping platforms. Front. Plant Sci. 14, 1233794. doi: 10.3389/fpls.2023.1233794. PMID: 37680357 PMC10481964

[B32] Qi C. R. Su H. Mo K. Guibas L. J. (2017). PointNet++: Deep hierarchical feature learning on point sets in a metric space. In Guyon I. Luxburg U. V. Bengio S. Wallach H. Fergus R. Vishwanathan S. Garnett R. , eds. Advances in Neural Information Processing Systems 30 (NIPS 2017) (: Curran Associates, Inc.), 5099–5108. Available online at: https://proceedings.neurips.cc/paper/2017/hash/d8bf84be3800d12f74d8b05e9b89836f-Abstract.html (Accessed April 30, 2026).

[B33] Reji J. Nidamanuri R. R. (2024). Deep learning-based prediction of plant height and crown area of vegetable crops using LiDAR point cloud. Sci. Rep. 14, 14903. doi: 10.1038/s41598-024-65322-8. PMID: 38942825 PMC11213942

[B34] Schönberger J. L. Frahm J. M. (2016). Structure-from-motion revisited. In Proceedings of the IEEE Conference on Computer Vision and Pattern Recognition (CVPR) (Las Vegas, NV: IEEE Computer Society), 4104–4113. doi: 10.1109/CVPR.2016.406

[B35] Sun W. Li J. (2024). FPN-Point: Feature pyramid enhanced point cloud network for high-throughput plant seedling segmentation. Comput. Electron. Agric. 218, 108652.

[B36] Tang Z. He X. Zhou G. Chen A. Wang Y. Li L. . (2023). A precise image-based tomato leaf disease detection approach using PLPNet. Plant Phenomics 5, 42. doi: 10.34133/plantphenomics.0042. PMID: 37228516 PMC10204740

[B37] Vaswani A. Shazeer N. Parmar N. Uszkoreit J. Jones L. Gomez A. N. . (2017). Attention is all you need. Adv. Neural Inf. Process. Syst. 30. doi: 10.48550/arXiv.1706.03762

[B38] Wang P. Liu Y. Li Y. Fu X. (2023). Global-local feature fusion for point cloud semantic segmentation. Pattern Recognit. 140, 109345. doi: 10.1016/j.patcog.2023.109345. PMID: 38826717

[B39] Wang Y. Sun Y. Liu Z. Sarma S. E. Bronstein M. M. Solomon J. M. (2019). Dynamic graph CNN for learning on point clouds. ACM Trans. Graphics 38, 1–12. doi: 10.1145/3326362

[B40] Yang W. Feng H. Zhang X. Zhang J. Doonan J. H. Batchelor W. D. . (2020). Crop phenomics and high-throughput phenotyping: Past decades, current challenges, and future perspectives. Mol. Plant 13, 187–214. doi: 10.1016/j.molp.2020.01.008. PMID: 31981735

[B41] Yang F. Zhang Y. Liu X. Fu X. (2024). Coordinate calibration for plant point clouds based on RANSAC and planar constraints. J. Comput. Biol. Bioinf. 4, 1–8. doi: 10.1109/JCBB.2024.3389214. PMID: 25079929

[B42] Yao Y. Luo Z. Li S. (2018). MVSNet: Depth inference for unstructured multi-view stereo. In Ferrari V. Hebert M. Sminchisescu C. Weiss Y. , eds. Computer Vision – ECCV 2018: 15th European Conference, Munich, Germany, September 8-14, 2018, Proceedings, Part VIII ( Springer International Publishing) 11212, 785–801. doi: 10.1007/978-3-030-01237-3_47. Available online at: https://proceedings.neurips.cc/paper/2018/hash/7bccfde7714a1ebadf06c5f4cea77e44-Abstract.html (Accessed April 30, 2026).

[B43] Yuan X. Liu J. Wang H. Zhang Y. Tian R. Fan X. (2024). Prediction of useful eggplant seedling transplants using multi-view images. Agronomy 14, 2016. doi: 10.3390/agronomy14092016. PMID: 30654563

[B44] Zabawa M. Stavness I. Popović M. (2021). “ Automated surface area estimation of plants based on 3D point clouds”, in: Proceedings of the Computer Vision for Plant Phenotyping Workshop (CVPPA). doi: 10.1109/CVPPA52837.2021.9575458

[B45] Zermas D. Morellas V. Mulla D. Papanikolopoulos N. (2020). 3D model processing for high throughput phenotype extraction – the case of corn. Comput. Electron. Agric. 172, 105047. doi: 10.1016/j.compag.2019.105047. PMID: 38826717

[B46] Zhang Y. Li J. Wang Y. Liu H. (2024). Three-dimensional phenotyping pipeline of potted plants based on neural radiation fields and path segmentation. Plants 13, 3368. doi: 10.3390/plants13193368. PMID: 39683161 PMC11644607

[B47] Zhang W. Wu S. Wen W. Lu X. Wang C. Gou W. . (2023). Three-dimensional branch segmentation and phenotype extraction of maize tassel based on deep learning. Plant Methods 19, 76. doi: 10.1186/s13007-023-01051-9. PMID: 37528454 PMC10394845

[B48] Zhang H. Yang J. Zhao Y. (2023). MS-PCNet: Multi-scale point cloud fusion network for agricultural small target segmentation. Biosyst. Eng. 229, 104–118.

[B49] Zhao J. Chen Z. Yang S. Fu X. (2024). Semantic-guided density clustering for high-precision plant instance segmentation. Pattern Recognit. Lett. 177, 1–7. doi: 10.1016/j.patrec.2024.03.012. PMID: 38826717

